# Karyological characterization and identification of four repetitive element groups (the 18S – 28S rRNA gene, telomeric sequences, microsatellite repeat motifs, *Rex* retroelements) of the Asian swamp eel (*Monopterus albus*)

**DOI:** 10.3897/CompCytogen.v11i3.11739

**Published:** 2017-06-22

**Authors:** Aorarat Suntronpong, Watcharaporn Thapana, Panupon Twilprawat, Ornjira Prakhongcheep, Suthasinee Somyong, Narongrit Muangmai, Surin Peyachoknagul, Kornsorn Srikulnath

**Affiliations:** 1 Laboratory of Animal Cytogenetics and Comparative Genomics (ACCG), Department of Genetics, Faculty of Science, Kasetsart University, 50 Ngamwongwan, Chatuchak, Bangkok 10900, Thailand; 2 Animal Breeding and Genetics Consortium of Kasetsart University (ABG – KU), 50 Ngamwongwan, Chatuchak, Bangkok 10900, Thailand; 3 National Center for Genetic Engineering and Biotechnology (BIOTEC), 113 Thailand Science Park, Phaholyothin Road, Klong Nueng, Klong Luang, Pathum Thani 12120, Thailand; 4 Department of Fishery Biology, Faculty of Fisheries, Kasetsart University, 50 Ngamwongwan, Chatuchak, Bangkok 10900, Thailand; 5 Center for Advanced Studies in Tropical Natural Resources, National Research University-Kasetsart University, Kasetsart University, Thailand (CASTNAR, NRU-KU, Thailand), Kasetsart University, Bangkok 10900, Thailand; 6 Department of Biology, Faculty of Science, Naresuan University, Phitsanulok 65000, Thailand

**Keywords:** Asian swamp eel, C-band, dispersion, microsatellite repeat, retroelement

## Abstract

Among teleost fishes, Asian swamp eel (*Monopterus albus* Zuiew, 1793) possesses the lowest chromosome number, 2n = 24. To characterize the chromosome constitution and investigate the genome organization of repetitive sequences in *M. albus*, karyotyping and chromosome mapping were performed with the 18S – 28S rRNA gene, telomeric repeats, microsatellite repeat motifs, and *Rex* retroelements. The 18S – 28S rRNA genes were observed to the pericentromeric region of chromosome 4 at the same position with large propidium iodide and C-positive bands, suggesting that the molecular structure of the pericentromeric regions of chromosome 4 has evolved in a concerted manner with amplification of the 18S – 28S rRNA genes. (TTAGGG)n sequences were found at the telomeric ends of all chromosomes. Eight of 19 microsatellite repeat motifs were dispersedly mapped on different chromosomes suggesting the independent amplification of microsatellite repeat motifs in *M. albus*. *Monopterus albus Rex1* (*MALRex1*) was observed at interstitial sites of all chromosomes and in the pericentromeric regions of most chromosomes whereas *MALRex3* was scattered and localized to all chromosomes and *MALRex6* to several chromosomes. This suggests that these retroelements were independently amplified or lost in *M. albus*. Among *MALRexs* (*MALRex1*, *MALRex3*, and *MALRex6*), *MALRex6* showed higher interspecific sequence divergences from other teleost species in comparison. This suggests that the divergence of *Rex6* sequences of *M. albus* might have occurred a relatively long time ago.

## Introduction

Teleost fishes possess high morphological and physiological variation with nearly 30,000 extant species (Nelson 2016). The Asian swamp eel (*Monopterus albus* Zuiew, 1793) is a commercially important, air-breathing fish (Synbranchidae, Synbranchiformes) which is a protogynous hermaphrodite native in freshwaters of East and Southeast Asia and invasive elsewhere in the world including North America (Liem 1963, Chan et al. 1972, Cheng et al. 2003). The diploid chromosome number of *M. albus* is 24, comprising 12 pairs of acrocentric chromosomes (Yu et al. 1989, Ji et al. 2003). This is considered to be the lowest chromosome number known in teleosts (genome sizes 0.6–0.8 pg), while common chromosome numbers of teleosts are 2n = 40–50 and genome sizes around 0.8–2 pg (Zhou et al. 2002). The Asian swamp eel is, therefore, a good model to investigate genome evolution and the developmental process in teleosts.

Synbranchids are freshwater eel-like fishes which include four genera (*Macrotrema* Cantor, 1849, *Monopterus* Lacépède, 1800, *Ophisternon* McClelland, 1844, and *Synbranchus* Bloch, 1795) and *Monopterus* is phylogenetically located at the basal position except for the *Macrotrema* (Perdices et al. 2005, Betancur et al. 2013). This phylogenetic relationship suggests that the Asian swamp eel might retain the ancestral karyotype of Synbranchidae. When compared to other synbranchids, it has a unique karyotype with very few chromosomes. For example, the diploid chromosome numbers of *Monopterus cuchia* Hamilton, 1822, a closely related species, is 42 and those of *Synbranchus* and *Ophisternon* species are 42 and 46, respectively (Rishi and Haobam 1984, Foresti et al. 1992, Nirchio et al. 2011, Carvalho et al. 2012, Utsunomia et al. 2014). An investigation of *M. albus* chromosome constitution to compare it with other synbranchid fishes could shed light evolutionary scenarios of chromosomal rearrangements and genome organization within Synbranchidae.

Vertebrate genomes are commonly characterized by a large copy number of repetitive sequences, belonging to two main classes: the site-specific type (such as satellite DNA, microsatellite repeats, ribosomal RNA genes and telomeric sequences), and the interspersed type (transposable elements, TEs) (Jelinek and Schmid 1982). Although most repetitive DNAs do not code for proteins, repetitive sequences can also play important role in the function, dynamics, and evolution of genomes (Csink and Henikoff 1998, Henikoff et al. 2001). Microsatellites, which are tandem repeats of small stretches of DNA motifs, are widespread in the genomes. Amplification of microsatellite repeat motifs has often been observed on sex chromosomes (Cioffi et al. 2011, Matsubara et al. 2015) or several autosomes (Schneider et al. 2015) of vertebrates. Microsatellite repeat motifs have been widely used as cytogenetic markers for chromosome identification, particularly for map-poor species (Srikulnath 2010). TEs are also thought to play an important role in genome evolution (Kidwell and Lisch 2000) acting as a substrate for homologous recombination resulting in chromosomal rearrangements. Additionally, TEs can be transmitted by both vertical and horizontal transfers being present in genomes of phylogenetically distant species (Tang et al. 2015). Retrotransposons (retroelements) are a class of TEs which have RNA as an intermediate, and the *Rex* retroelements (*Rex1*, *Rex3*, and *Rex6*) were active during teleost evolution (Volff et al. 1999, 2000, 2001). These retroelements are widely used as markers for molecular evolution and physical mapping, which allow to understand the role of repetitive elements in genome organization and evolution of teleosts (Ferreira et al. 2011, Schneider et al. 2013).

In this study, karyotyping was performed with conventional Giemsa staining, 4', 6-diamidino-2-phenylindole (DAPI) and propidium iodide (PI) fluorescent staining, C-banding, and fluorescence *in situ* hybridization (FISH) with four repetitive elements; namely, the 18S − 28S ribosomal RNA genes, telomeric (TTAGGG)n sequences, *Rex* retroelements and 19 microsatellite repeat motifs. Partial DNA fragments of *Rex* retroelements (*Rex1*, *Rex3*, and *Rex6*) were molecularly characterized and the evolutionary processes responsible for these retroelements in teleost genomes were discussed, together with the organization of synbranchid genomes.

## Materials and methods

### Specimens and chromosome preparation

Ten specimens of the Asian swamp eel were purchased from an animal pet shop in Bangkok, Thailand. Animal care and all experimental procedures were approved by the Animal Experiment Committee, Kasetsart University, Thailand (approval no. ACKU00958), and conducted according to the Regulations on Animal Experiments at Kasetsart University, Thailand. Mitotic chromosomes were obtained from gill and kidney cells using the air drying method. Briefly, after intraperitoneal injection of 0.01% colchicine (Sigma, St. Louis, Missouri, USA) in the proportion of 0.7 ml per 100 g of fish weight for 2 h, fishes were anesthetized in ice-cold water, and the anterior portion of the gill and kidney were removed and used for mitotic chromosome preparation. After hypotonic treatment of gill and kidney in 0.075 M KCl for 50 min at room temperature, the organs were minced and placed in the first fixative solution (3:1 methanol/acetic acid) for 5 min and in the second fixative solution (2:1 methanol/acetic acid) for 5 min on ice. The cells were collected by filtration using gauze, and then fixed with 3:1 methanol/acetic acid. The cells in suspension were dropped onto clean glass slides and air-dried. The slides were kept at -80°C until use. For karyotyping with conventional Giemsa staining, the chromosome slides were stained with 4% Giemsa solution (pH 7.2) for 10 min.

### C-banding

To examine the chromosomal distribution of constitutive heterochromatin, C-banding was performed using the standard barium hydroxide/saline/Giemsa method (Sumner 1972) with slight modification as follows: chromosome slides were treated with 0.2 N HCl at room temperature for 60 min and then with 5% Ba(OH)_2_ at 50°C for 15 s, followed by 2× SSC at 65°C for 60 min.

### Polymerase chain reaction (PCR) amplification and molecular cloning

Genomic DNA was extracted from liver and muscle tissue following the standard salting-out protocol as described previously (Supikamolseni et al. 2015), and used as templates for polymerase chain reaction (PCR). Partial DNA fragments of the 18S − 28S rRNA genes, and *Rex* retroelements (*Rex1*, *Rex3*, and *Rex6*) were amplified using following PCR primers (see Suppl. material 1). PCR amplification was performed using 20 μl of 1× ExTaq buffer containing 1.5 mM MgCl_2_, 0.2 mM dNTPs, 5.0 μM the primers, and 0.25 U of TaKaRa Ex Taq (TaKaRa Bio, Otsu, Japan), and 25 ng of genomic DNA. PCR conditions were as follows: an initial denaturation at 94°C for 3 min, followed by 35 cycles of 94°C for 30 s, 53–59°C for 30 s, and 72°C for 45 s, and a final extension at 72°C for 10 min. The PCR products were cloned using the pTG19-T vector (Vivantis Technologies Sdn Bhd, Selangor Darul Ehsan, Malaysia), and nucleotide sequences of the DNA fragments were determined using DNA sequencing service (First BASE Laboratories Sdn Bhd, Seri Kembangan, Selangor, Malaysia). Nucleotide sequences of three to five DNA clones, and their consensus sequences were searched for homologies with annotated sequences in the National Center for Biotechnology Information (NCBI) database to identify the amplified DNA fragments, using the BLASTx and BLASTn programs (http://blast.ncbi.nlm.nih.gov/Blast.cgi). They were then deposited in the DNA Data Bank of Japan (DDBJ; http://www.ddbj.nig.ac.jp/index-e.html) (Suppl. material 2).

### Sequence analysis

Multiple sequence alignments of the three data sets (*Rex1*, *Rex3*, and *Rex6*) were performed with those of other teleosts taken from the NCBI database (Suppl. material 2), using the default parameters of Molecular Evolutionary Genetics Analysis 6 (MEGA6) software (Center for Evolutionary Functional Genomics, The Biodesign Institute, Tempe, AZ, USA) (Tamura et al. 2013). Numbers of indels (insertions and deletions) for each data set of *Rex* retroelements were calculated using the multiallelic mode of DNAsp 5.0 (Librado and Rozas 2009). All unalignable and gap-containing sites were carefully removed from the data sets. Interspecific sequence divergence was estimated using uncorrected pairwise distances (*p*-distances), and for the *Rex* reverse transcriptase region, synonymous (K_s_) and nonsynonymous (K_a_) substitution rates (±standard error) were calculated using the Nei-Gojobori method (Nei and Gojobori 1986) with Jukes-Cantor correction (Jukes and Cantor 1969). Phylogenetic analyses were then performed using Bayesian Inference (BI) using MrBayes v3.0b4 (Huelsenbeck and Ronquist 2001) and the optimal model of DNA substitution was determined for each data set using Kakusan4 (Tanabe 2011). The Markov Chain Monte Carlo (MCMC) process was set to run four chains simultaneously for one million generations. After the log-likelihood value plateaued, a sampling procedure was performed every 100 generations to obtain 10,000 trees, and subsequently to provide a majority-rule consensus tree with average branch lengths. All sample points were discarded as burn-in prior to reaching convergence, and the Bayesian posterior probability in the sampled tree population was obtained in percentage terms. All phylogenetic trees were midpoint-rooted due to the absence of suitable outgroup in *Rex3* data set. However, additional phylogenetic tree based on *Rex1* and *Rex6* sequences were constructed with using outgroup method from other *Rex* sequences.

### FISH mapping

Chromosomal locations of the 18S – 28S rRNA genes, *Rex* retroelements (*Rex1*, *Rex3*, and *Rex6*), telomeric (TTAGGG)n sequences, and 19 microsatellite repeat motifs: (CA)_15_, (GC)_15_, (GA)_15_, (AT)_15_, (CAA)_10_, (CAG)_10_, (CAT)_10_, (CGG)_10_, (GAG)_10_, (AAT)_10_, (AAGG)_8_, (AATC)_8_, (AGAT)_8_, (ACGC)_8_, (AAAT)_8_, (AAAC)_8_, (AATG)_8_, (AAATC)_6_, and (AAAAT)_6_ were determined using FISH, as described previously (Matsuda and Chapman 1995, Srikulnath et al. 2009). We used a 1,366-bp genomic DNA fragment of *M. albus* 18S – 28S rRNA genes (LC151290), a 533-bp genomic DNA fragment of *M. albus Rex1* (LC110446), a 415-bp genomic DNA fragment of *M. albus Rex3* (LC110447), a 471-bp genomic DNA fragment of *M. albus Rex6* (LC110448), biotin-labeled 42-bp TTAGGG repeat, and 19 biotin-labeled oligonucleotide microsatellite repeat probes, respectively. We labeled 250 ng of DNA fragments with biotin-16-dUTP (Roche Diagnostics, Mannheim, Germany) by nick translation, according to the manufacturer’s protocol and ethanol-precipitated with salmon sperm DNA and *Escherichia coli* tRNA. After hybridization of biotin-labeled probes to *M. albus* chromosomes, the probes were stained with avidin labeled with fluorescein isothiocyanate (avidin-FITC; Invitrogen, CA, USA). Slides were subsequently stained with 0.75 μg/ml PI or 1 µg/ml DAPI. Fluorescence hybridization signals were captured using a cooled CCD camera mounted on a ZEISS Axioplan2 microscope and processed using MetaSystems ISIS v.5.2.8 software (MetaSystems, Alltlussheim, Germany).

For dual-color FISH, two probes differentially labeled with either biotin-16-dUTP or digoxigenin-11-dUTP (Roche Diagnostics) were mixed in hybridization buffer and co-hybridized to one slide. After hybridization, digoxigenin- and biotin-labeled probes were stained with anti-digoxigenin-rhodamine Fab fragments (Roche Diagnostics) and avidin labeled with fluorescein isothiocyanate (avidin-FITC; Invitrogen), respectively.

## Results

### Karyotype of *Monopterus albus*

**Figure 1.**
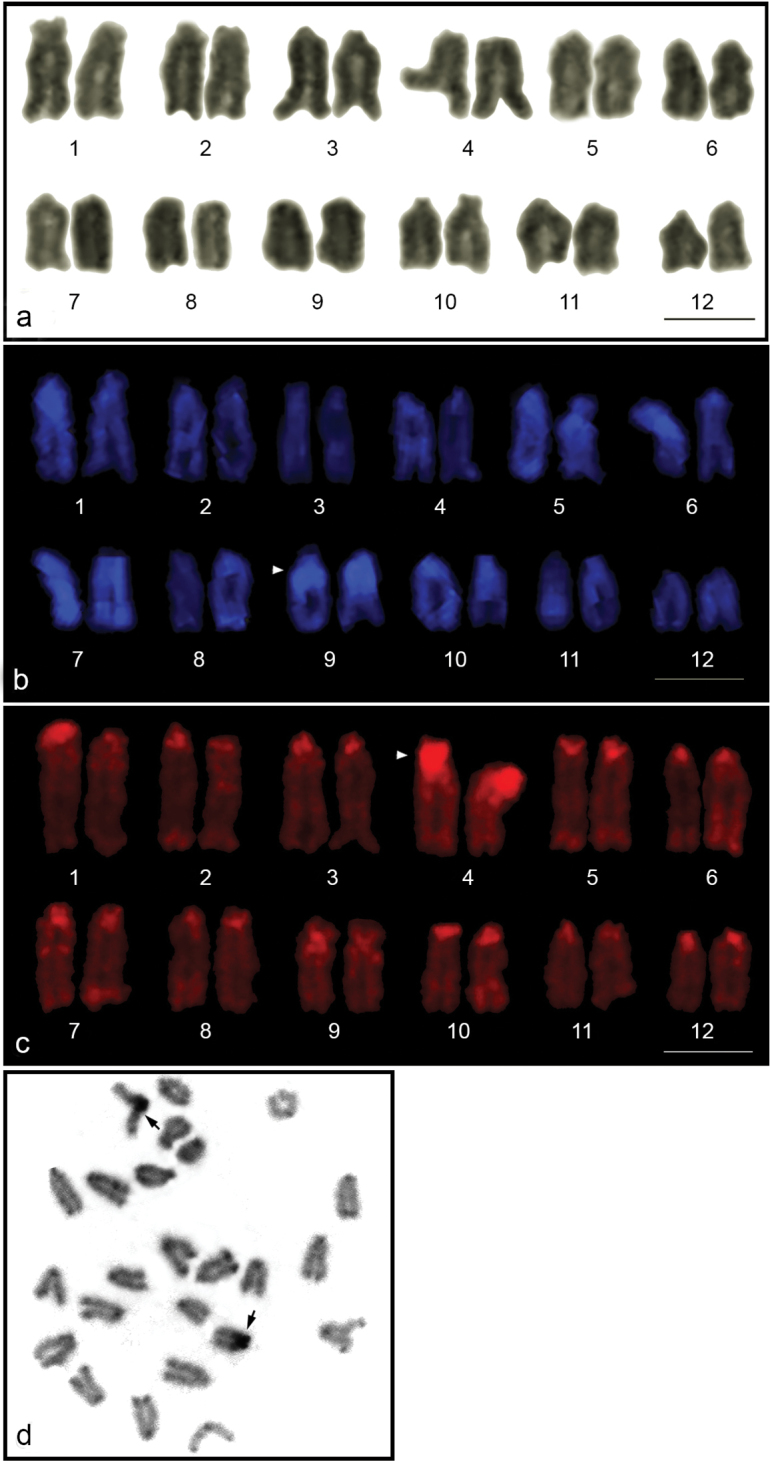
Giemsa-stained (**a**), DAPI-stained (**b**), PI-stained karyotype (**c**), and C-banded metaphase spread (**d**) of *Monopterus albus*. Arrowheads indicate the large DAPI-stained and large PI-stained regions. Arrows indicate C-positive heterochromatin blocks. Scale = 10 μm.

Over 10 Giemsa-stained metaphase spreads were examined for each *M. albus* individual. Diploid chromosome number is 24 (FN = 24) comprising twelve pairs of acrocentric chromosomes (Fig. 1a). The size difference of chromosome pairs was sequential, but most pairs were identified by size and banding pattern with DAPI and PI fluorescent staining. Large DAPI-positive bands were observed at the pericentromeric region of chromosome 9 (Fig. 1b), and large PI-positive bands were found at the pericentromeric region of chromosome 4 (Fig. 1c) coincident with a large C-positive heterochromatin bands (Fig. 1d).

### Chromosomal location of the 18S – 28S rRNA genes and (TTAGGG)_n_ sequences

**Figure 2.**
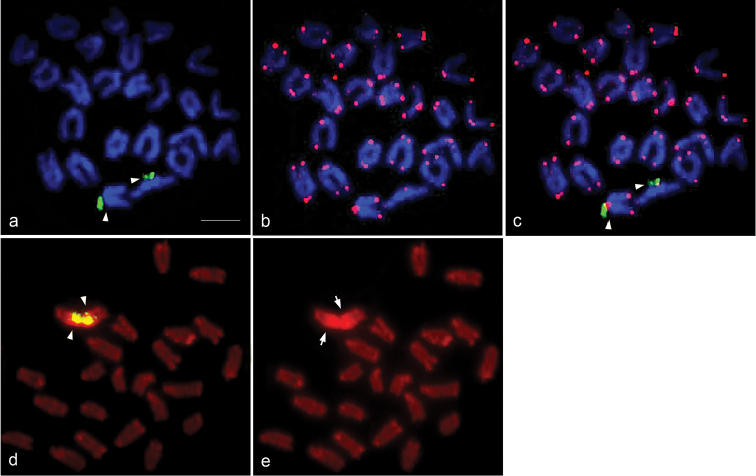
Chromosomal locations of the 18S – 28S rRNA genes and (TTAGGG)n sequences in *Monopterus albus*. Hybridization pattern of FITC-labeled 18S – 28S rRNA genes (green) (**a**) and rhodamine-labeled TTAGGG repeats (red) (**b**) on DAPI-stained chromosomes, and their co-hybridization pattern (**c**). Hybridization pattern of FITC-labeled 18S – 28S rRNA genes (green) (**d**) on PI-stained chromosomes. PI-stained patterns of the same metaphase spreads of (**d**) is shown in (**e**). Arrowheads indicate FISH signals of the 18S – 28S rRNA genes. Arrows indicate the large PI-stained region. Scale =10 μm.

Fluorescence hybridization signals for the 18S – 28S rRNA genes were also detected at the pericentromeric region of chromosome 4 co-localizing with both PI-positive bands and large C-positive heterochromatin blocks (Fig. 2a, c, d, e). Hybridization signals of TTAGGG repeats were observed at telomeric ends of all chromosomes, but no interstitial signal was found (Fig. 2b, c).

### Chromosomal localization of microsatellite repeat motifs

**Figure 3.**
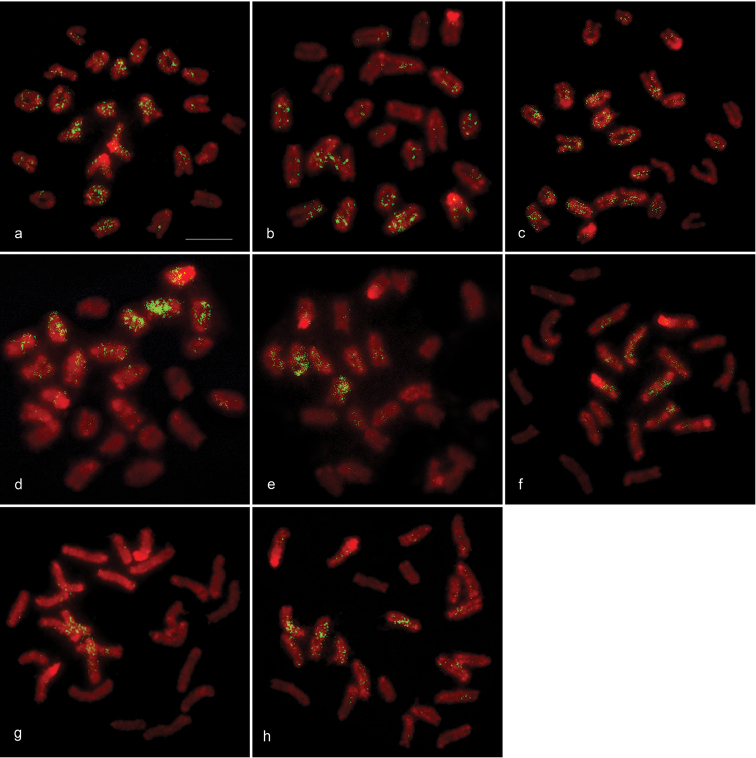
Chromosomal locations of microsatellite repeat motifs in *Monopterus albus*. Hybridization pattern of FITC-labeled (CAA)_10_ (**a**), (CAG)_10_ (**b**), (CGG)_10_ (**c**), (GAG)_10_ (**d**), (AGAT)_8_ (**e**), (ACGC)_8_ (**f**), (AAAT)_8_ (**g**), and (AAATC)_6_ (**h**) on PI-stained chromosomes.

Eight of the 19 microsatellite repeat motifs were dispersedly mapped onto most chromosomes (Fig. 3). Notably, strong hybridization signals of trinucleotide (CGG)_10_ were localized to chromosomes 2, 4, and 6, tetranucleotide (AAAT)_8_ to chromosomes 3 and 5, (AGAT)_8_ to chromosomes 5 and 9, (ACGC)_8_ to chromosomes 1, 2, 4, 7, 8 and 9, and pentanucleotide (AAATC)_6_ to chromosomes 1 and 8. No signal was observed from the other 11 microsatellite repeat motifs ((CA)_15_, (GC)_15_, (GA)_15_, (AT)_15_, (CAA)_10_, (CAG)_10_, (CAT)_10_, (GAG)_10_, (AAT)_10_, (AAGG)_8_, (AATC)_8_, (AAAC)_8_, (AATG)_8_, and (AAAAT)_6_).

### Chromosomal distribution of *Rex* retroelements (*Rex1*, *Rex3*, and *Rex6*)

**Figure 4.**
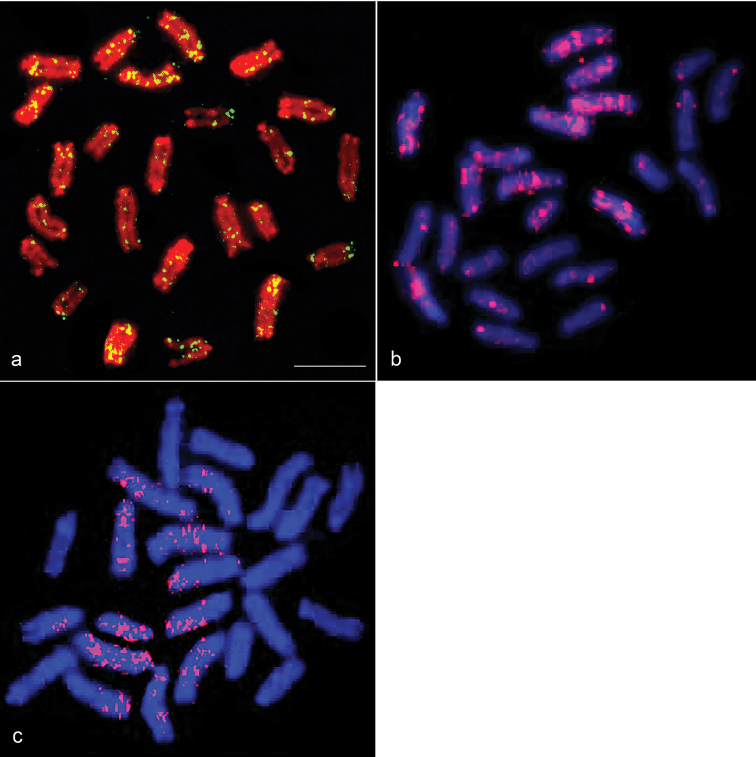
Chromosomal locations of *Rex1*, *Rex3*, and *Rex6* in *Monopterus albus*. Hybridization pattern of FITC-labeled *Rex1* (green) (**a**) on PI-stained chromosomes, and rhodamine-labeled *Rex3* (red) (**b**) and *Rex6* (red) (**c**) on DAPI-stained chromosomes. Scale =10 μm.

**Figure 5.**
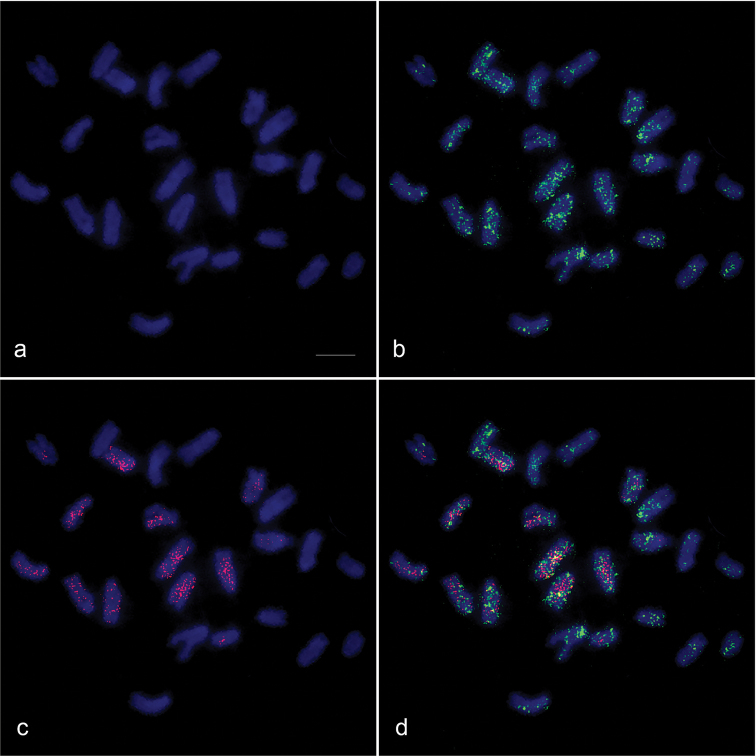
Chromosomal locations of *Rex3* and *Rex6* in *Monopterus albus*. Hybridization pattern of FITC-labeled *Rex3* (green) (**b**) and rhodamine-labeled *Rex6* (red) (**c**) on DAPI-stained chromosomes, and their co-hybridization pattern (**d**). DAPI-stained patterns of the same metaphase spreads of (**b, c**, and **d**) is shown in (**a**). Scale =10 μm.

*M. albus Rex1* (*MALRex1*) obtained from a single *M. albus* individual was localized to the pericentromeric region and interstitial sites of all chromosomes, except for chromosomes 4 and 9 where *MALRex1* was found only at interstitial sites (Fig. 4a). *MALRex3* was located scattered in all chromosomes with strong hybridization signals observed on chromosomes 1–4 and 8 and weak signals on chromosomes 5–7 and 9–12 (Figs 4b, 5b, d). FISH signals of *MALRex6* were found on chromosomes 1, 2, 5, 6, 8, and 10 as dispersion along the chromosomes (Figs 4c, 5c, d).

### Molecular evolutionary dynamics of *Rex* retroelements

**Figure 6.**
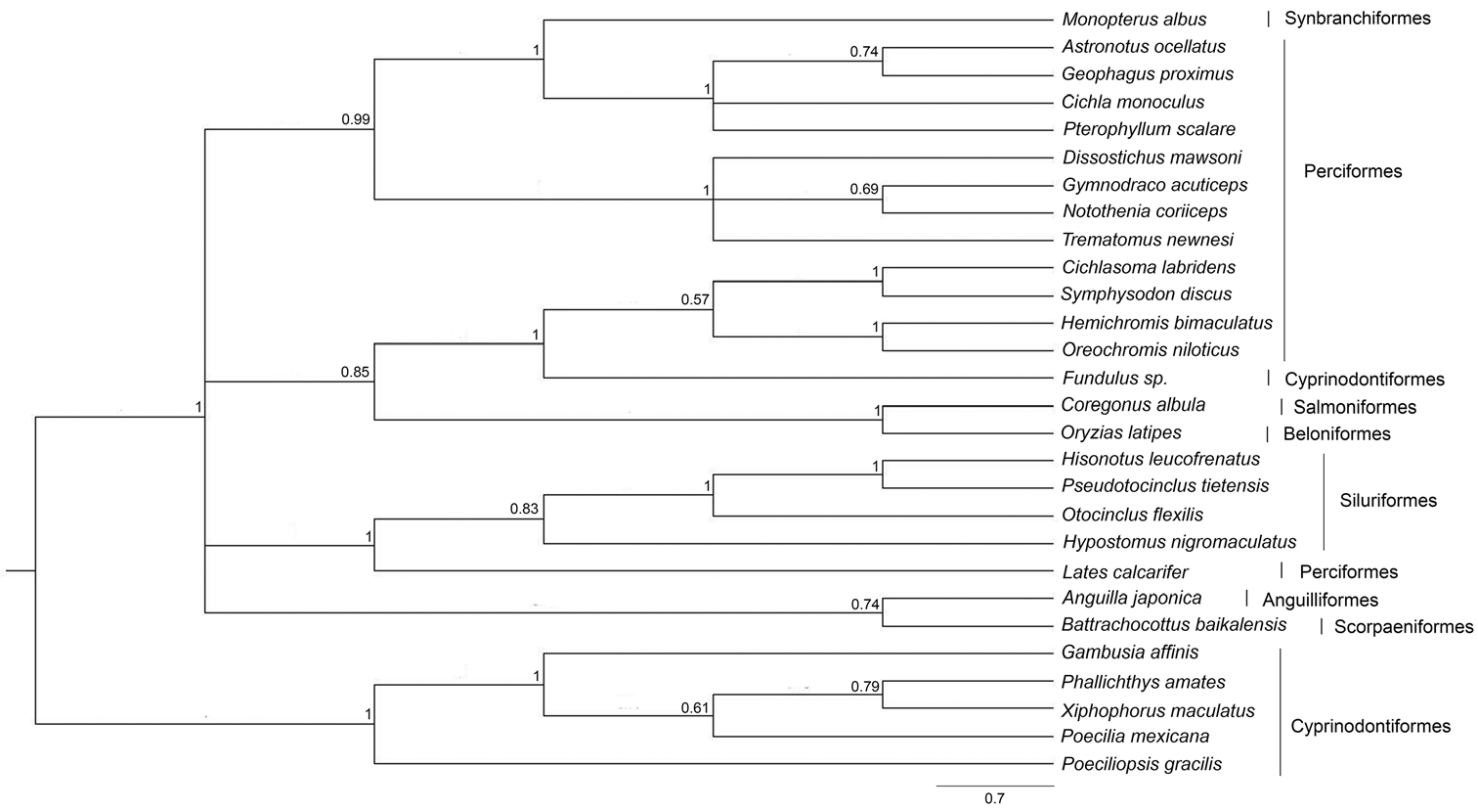
Phylogenetic placements of partial nucleotide sequences of *Rex1* from 28 teleosts. Support values at each node are Bayesian posterior probability.

**Figure 7.**
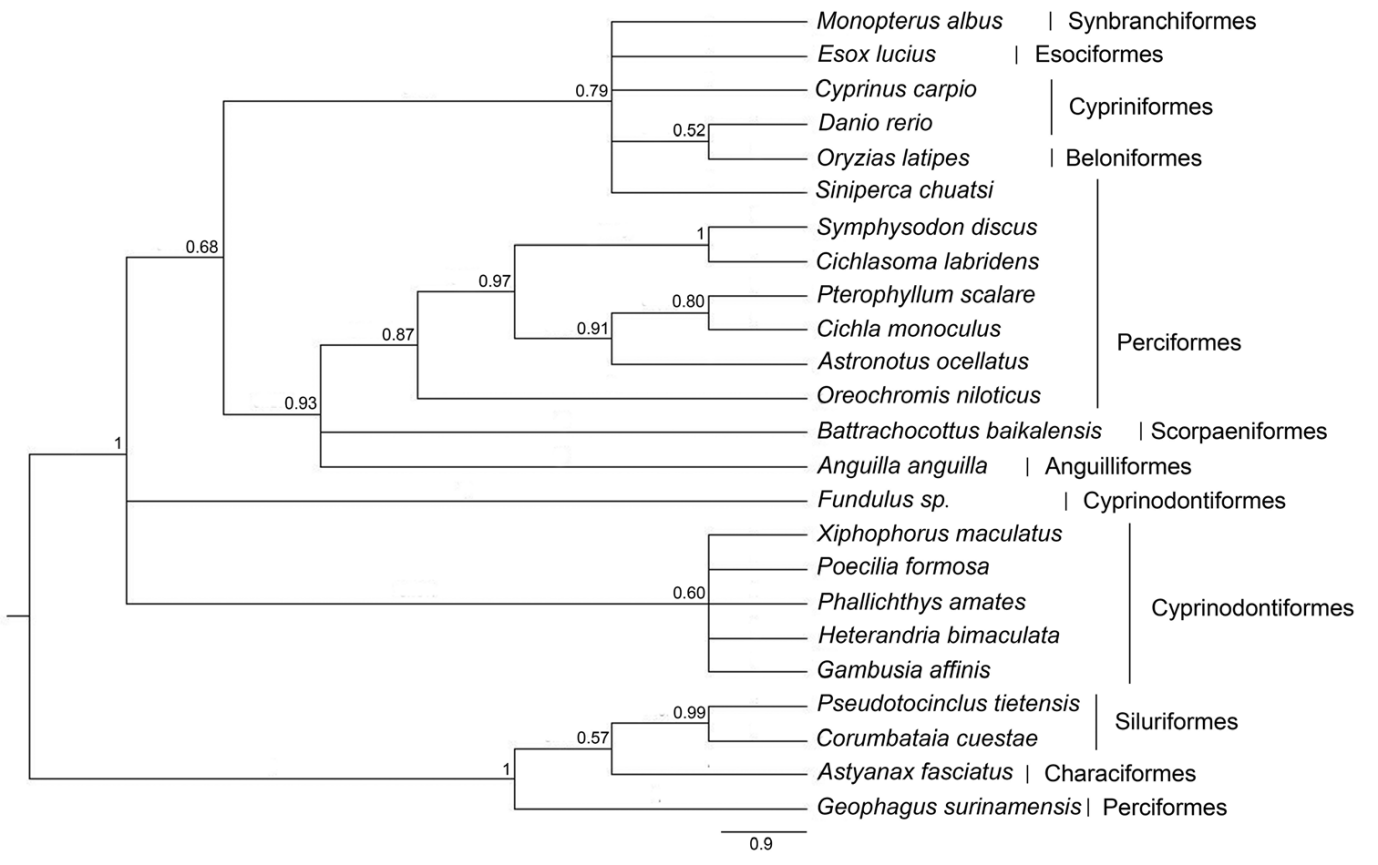
Phylogenetic placements of partial nucleotide sequences of *Rex3* from 24 teleosts. Support values at each node are Bayesian posterior probability.

**Figure 8.**
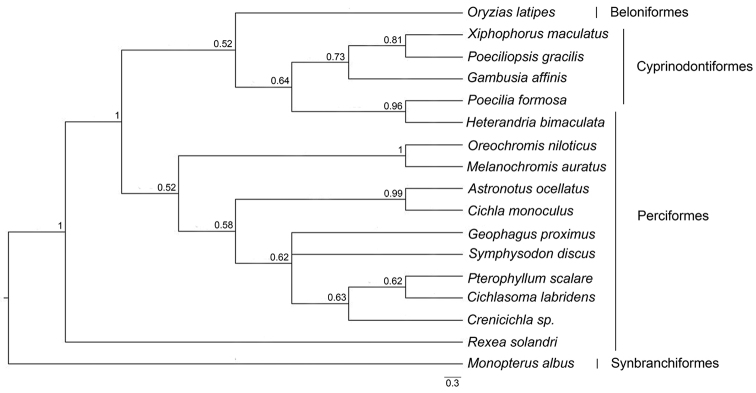
Phylogenetic placements of partial nucleotide sequences of *Rex6* from 17 teleosts. Support values at each node are Bayesian posterior probability.

The nucleotide sequence of a 533 bp-fragment of *MALRex1* was used in multiple sequence alignment with 28 other teleosts, evidencing 32 indel sites. Sequence divergence among species varied from 0 to 50.13% with an average of 29.56±1.13% (Suppl. material 3). *MALRex1* sequences in *M. albus* showed the minimum interspecific sequence divergence of 1.88% from nototheniids *Dissostichus mawsoni* Norman, 1937 and *Notothenia coriiceps* Richardson, 1844 (Perciformes) and the maximum divergence of 41.95% to *Poeciliopsis gracilis* Heckel, 1848 (Cyprinodontiformes); the average is 24.51±8.14%. The phylogenetic placement of *Rex1* sequences showed that most species were grouped in their respective orders (Fig. 6, Suppl. material 6). The average K_s_/K_a_ value of *Rex1* sequences was 2.19±0.08 (Table 1). The nucleotide sequence of a 415 bp-fragment of *MALRex3* was used in multiple sequence alignment with 24 other teleosts, showing 23 indels. The average sequence divergence among species was 33.94±17.24%, ranging from 2.65% to 69.54% (Suppl. material 4). *MALRex3* sequences showed the minimum interspecific sequence divergence of *M. albus*, 18.54%, from *Esox lucius* Linnaeus, 1758 (Esociformes) and the maximum divergence, 66.65%, from *Astyanax fasciatus* Cuvier, 1819 (Characiformes); average 31.84±12.74%. The phylogenetic placement of *Rex3* sequences showed a clade for each order except for Perciformes fishes (Fig. 7). The average K_s_/K_a_ value of *Rex3* sequences was 1.05±0.05 (Table 2). The nucleotide sequences of a 471 bp-fragment of *MALRex6* was used in multiple sequence alignment with 17 other teleosts showing 15 indels. The sequence divergences among species varied from 3.13 to 65.546% (average 27.94±19.53%). *MALRex6* sequences showed the minimum interspecific sequence divergence of *M. albus*, 60.31%, from *Geophagus proximus* Castelnau, 1855 (Perciformes) and the maximum divergence, 65.54%, from *Oreochromis niloticus* Cuvier, 1832 (Perciformes,); average 62.60±1.14% (Suppl. material 5). The phylogenetic placement of *Rex6* sequences showed a clade for each order (Fig. 8, Suppl. material 7). The average K_s_/K_a_ value of *Rex6* sequences was 0.85±0.04 (Table 3).

**Table 1.** Synonymous substitution site (K_s_) per nonsynonymous substitution sites (K_a_) of *Rex1* retroelement among twenty eight teleosts.

	AJA	PTI	HLE	HNI	OFL	CAL	OLA	FUN	GAF	PME	PAM	PGR	XMA	MAL	LCA	AOC	CMO	CLA	GPR	HBI	ONI	PSC	SDI	DMA	NCO	TNE	GAC	BBA
*Anguilla japonica* (AJA)																												
*Pseudotocinclus tietensis* (PTI)	2.09																											
*Hisonotus leucofrenatus* (HLE)	1.97	2.97																										
*Hypostomus nigromaculatus* (HNI)	2.71	3.07	3.14																									
*Otocinclus flexilis* (OFL)	2.18	2.82	2.69	3.43																								
*Coregonus albula* (CAL)	3.20	3.28	2.78	2.75	3.22																							
*Oryzias latipes* (OLA)	1.93	2.37	2.14	2.67	2.19	2.62																						
*Fundulus* sp. (FUN)	2.05	2.06	1.76	2.38	2.39	2.15	1.62																					
*Gambusia affinis* (GAF)	1.75	1.87	1.62	1.81	1.97	2.20	1.56	1.74																				
*Poecilia mexicana* (PME)	2.06	1.83	1.79	1.76	1.70	2.33	1.62	1.83	4.03																			
*Phallichthys amates* (PAM)	1.82	1.52	1.51	1.80	1.79	2.01	1.53	1.71	2.22	3.31																		
*Poeciliopsis gracilis* (PGR)	1.55	1.67	1.57	1.72	1.81	1.64	1.71	1.53	2.43	2.10	2.17																	
*Xiphophorus maculatus* (XMA)	1.85	1.72	1.54	1.86	1.99	2.16	1.66	1.78	3.23	3.20	2.11	2.26																
*Monopterus albus* (MAL)	2.65	2.52	1.80	3.08	1.97	3.18	2.45	2.56	1.93	2.20	1.88	1.50	1.91															
*Lates calcarifer* (LCA)	1.34	2.05	1.44	3.01	2.60	2.45	2.21	1.65	1.62	1.84	1.64	1.44	1.75	1.75														
*Astronotus ocellatus* (AOC)	2.63	2.27	1.90	2.60	2.77	2.55	2.27	2.47	1.82	1.89	1.80	1.58	1.84	1.46	1.91													
*Cichla monoculus* (CMO)	2.69	2.30	1.93	2.65	2.83	2.60	2.30	2.43	1.82	1.89	1.80	1.60	1.84	1.49	1.91	0.00												
*Cichlasoma labridens* (CLA)	2.24	2.59	2.55	2.92	2.70	2.82	2.07	2.49	1.80	1.80	1.69	1.41	1.77	2.24	2.14	2.31	2.26											
*Geophagus proximus* (GPR)	2.63	2.27	1.90	2.60	2.77	2.55	2.27	2.47	1.82	1.89	1.80	1.58	1.84	1.46	1.91	n/c	0.00	2.31										
*Heterandria bimaculata* (HBI)	2.01	2.72	2.69	2.44	2.67	2.57	2.15	1.94	1.64	1.89	1.72	1.57	1.63	2.32	2.04	2.39	2.35	3.02	2.39									
*Oreochromis niloticus* (ONI)	1.82	2.60	2.44	2.86	2.67	2.59	2.11	2.35	1.70	1.91	1.73	1.64	1.68	2.24	2.02	2.35	2.30	3.80	2.35	1.86								
*Pterophyllum scalare* (PSC)	2.69	2.30	1.93	2.65	2.83	2.60	2.30	2.43	1.82	1.89	1.80	1.60	1.84	1.49	1.91	0.00	n/c	2.26	0.00	2.35	2.30							
*Symphysodon discus* (SDI)	2.13	2.55	2.47	2.74	2.57	2.63	2.08	2.43	1.72	1.89	1.74	1.58	1.68	2.02	2.02	2.06	2.02	1.35	2.06	2.37	3.56	2.02						
*Dissostichus mawsoni* (DMA)	2.86	2.58	1.99	2.83	2.23	3.34	2.16	2.05	1.64	1.96	1.67	1.52	1.70	1.89	1.90	2.20	2.24	2.40	2.20	2.00	2.22	2.24	2.23					
*Notothenia coriiceps* (NCO)	3.22	3.14	2.26	3.02	2.41	3.70	2.28	2.30	1.84	2.19	1.74	1.53	1.88	2.59	2.32	2.47	2.52	2.74	2.47	2.27	2.53	2.52	2.65	3.78				
*Trematomus newnesi* (TNE)	2.75	2.73	2.20	2.86	2.18	3.37	2.10	2.37	1.70	2.04	1.67	1.61	1.76	2.39	2.14	2.29	2.33	2.79	2.29	2.24	2.58	2.33	2.62	3.75	3.20			
*Gymnodraco acuticeps* (GAC)	2.71	2.67	2.00	2.84	2.09	3.18	2.10	2.04	1.71	1.98	1.63	1.54	1.75	2.06	2.16	2.16	2.20	2.41	2.16	2.03	2.25	2.20	2.27	1.76	1.79	1.45		
*Battrachocottus baikalensis* (BBA)	2.51	2.30	2.39	2.91	2.35	4.24	2.17	2.11	1.73	1.92	1.64	1.81	1.73	2.75	2.38	2.62	2.66	2.22	2.62	2.18	2.07	2.66	2.30	2.57	2.72	2.86	2.50	

n/c indicate that number is uncountable value.

**Table 2.** Synonymous substitution site (K_s_) per nonsynonymous substitution sites (K_a_) of *Rex3* retroelement among twenty four teleosts.

	AAN	CCA	DRE	AFA	CCU	PTI	ELU	OLA	FUN	GAF	HBI	PFO	PAM	XHE	MAL	SCH	AOC	CMO	CLA	GSU	ONI	PSC	SDI	BBA
*Anguilla anguilla* (AAN)																								
*Cyprinus carpio* (CCA)	1.40																							
*Danio rerio* (DRE)	1.33	1.56																						
*Astyanax fasciatus* (AFA)	1.02	1.07	0.90																					
*Corumbataia cuestae* (CCU)	0.94	1.16	0.97	1.62																				
*Pseudotocinclus tietensis* (PTI)	0.98	1.09	0.96	1.63	2.29																			
*Esox lucius* (ELU)	1.05	1.52	1.80	0.98	1.08	0.91																		
*Oryzias latipes* (OLA)	1.35	0.81	1.31	0.88	0.97	0.91	1.01																	
*Fundulus* sp.(FUN)	1.35	1.26	1.49	0.93	1.12	1.09	1.71	1.03																
*Gambusia affinis* (GAF)	1.13	0.84	1.19	0.88	1.09	0.96	1.11	0.81	0.80															
*Heterandria bimaculata* (HBI)	1.35	1.47	1.53	0.87	1.06	1.02	1.59	0.87	0.94	0.87														
*Poecilia formosa* (PFO)	1.19	0.94	1.45	0.83	1.01	0.96	1.15	1.19	0.91	0.83	1.07													
*Phallichthys amates* (PAM)	1.17	1.08	1.44	0.82	1.02	0.97	1.23	0.89	0.65	1.01	0.76	0.75												
*Xiphophorus hellerii* (XHE)	1.30	1.14	1.48	0.82	1.04	0.99	1.36	0.84	0.72	0.94	0.97	1.55	1.03											
*Monopterus albus* (MAL)	1.06	1.25	1.66	0.94	0.90	0.85	1.48	0.81	1.24	0.93	1.27	1.07	1.00	1.01										
*Siniperca chuatsi* (SCH)	1.12	0.95	1.36	0.90	0.96	0.97	0.97	0.63	1.54	0.97	1.32	1.28	1.02	1.22	0.92									
*Astronotus ocellatus* (AOC)	1.00	1.39	1.63	1.00	0.89	0.90	1.20	1.01	1.11	1.01	1.22	1.27	1.18	1.09	0.81	1.15								
*Cichla monoculus* (CMO)	0.92	0.95	1.28	0.99	1.01	0.98	0.92	0.75	0.85	0.84	0.89	0.87	0.79	0.74	0.71	0.74	1.03							
*Cichlasoma labridens* (CLA)	0.97	1.20	1.63	1.04	1.04	1.01	0.96	0.64	0.93	0.89	0.96	0.96	0.82	0.76	0.78	0.90	1.22	0.15						
*Geophagus surinamensis* (GSU)	1.02	1.13	1.07	1.50	1.84	2.27	1.15	0.96	1.15	1.07	1.10	1.10	1.07	1.05	0.92	1.01	0.94	1.04	1.06					
*Oreochromis niloticus* (ONI)	1.35	1.53	1.80	0.98	1.03	1.07	1.47	0.74	0.99	0.79	0.90	0.98	0.87	0.84	1.06	1.12	1.15	0.69	0.71	1.02				
*Pterophyllum scalare* (PSC)	1.24	1.56	1.37	1.01	0.88	0.90	1.55	1.04	1.22	1.11	1.02	1.25	1.25	1.17	0.98	1.21	1.65	1.51	1.26	0.97	0.99			
*Symphysodon discus* (SDI)	0.97	1.11	1.51	1.03	1.01	1.01	0.93	0.60	0.94	0.80	0.92	0.94	0.77	0.75	0.73	0.85	1.05	0.16	0.00	1.03	0.74	1.17		
*Battrachocottus baikalensis* (BBA)	1.16	0.92	1.42	0.98	0.94	0.93	0.93	0.70	1.07	0.89	0.92	0.87	0.74	0.78	0.84	0.54	0.91	0.70	0.77	1.07	0.71	1.06	0.70	

**Table 3.** Synonymous substitution site (K_s_) per nonsynonymous substitution sites (K_a_) of *Rex6* retroelement among seventeen teleosts.

	OLA	GAF	PFO	PGR	XMA	MAL	AOC	CLA	CMO	CRE	GPR	HBI	MAU	ONI	PSC	SDI	RSO
*Oryzias latipes* (OLA)																	
*Gambusia affinis* (GAF)	0.34																
*Poecilia formosa* (PFO)	0.28	0.71															
*Poeciliopsis gracilis* (PGR)	0.18	0.98	0.59														
*Xiphophorus maculatus* (XMA)	0.23	0.73	0.55	1.10													
*Monopterus albus* (MAL)	0.94	1.02	0.96	0.99	1.03												
*Astronotus ocellatus* (AOC)	0.71	0.68	0.64	0.60	0.62	0.88											
*Cichlasoma labridens* (CLA)	0.89	0.78	0.99	0.77	0.80	0.93	1.69										
*Cichla monoculus* (CMO)	0.55	0.42	0.54	0.41	0.33	0.86	0.90	1.35									
*Crenicichla* sp. (CRE)	0.33	0.40	0.49	0.33	0.29	0.85	0.51	1.18	0.73								
*Geophagus proximus* (GPR)	0.72	0.72	0.84	0.73	0.69	0.84	1.05	1.37	1.15	0.91							
*Hemichromis bimaculatus* (HBI)	0.27	1.16	0.00	0.85	0.82	0.95	0.74	1.02	0.59	0.57	0.90						
*Melanochromis auratus* (MAU)	0.64	0.58	0.68	0.67	0.59	0.81	0.83	1.20	1.32	0.66	1.14	0.79					
*Oreochromis niloticus* (ONI)	0.61	0.57	0.68	0.64	0.57	0.85	0.86	1.36	1.07	0.54	1.08	0.77	0.89				
*Pterophyllum scalare* (PSC)	0.75	0.73	0.91	0.73	0.69	0.90	1.03	1.83	1.54	1.20	1.33	1.00	1.27	1.01			
*Symphysodon discus* (SDI)	0.80	0.59	0.82	0.52	0.56	1.01	1.27	1.60	0.89	1.04	1.24	0.85	1.40	1.30	1.61		
*Rexea solandri* (RSO)	0.92	0.93	0.86	0.87	0.88	1.08	0.97	1.12	0.92	0.97	0.94	0.91	0.98	0.96	0.97	1.04	

## Discussion

### Karyotype and chromosomal localization of rRNA gene clusters, telomeric sequences, and microsatellite repeat motifs in *Monopterus albus*

The karyotype of *M. albus* (2n = 24, FN = 24) composed of 12 acrocentric chromosome pairs was found to be similar to that reported by Yu et al. (1989) and Ji et al. (2003). The chromosome number of *M. albus* is the lowest among synbranchids, e.g., *M. cuchia* (2n = 42, FN = 46) (Rishi and Haobam 1984), *Synbranchus marmoratus* Bloch, 1795 (2n = 42–46, FN = 46–54) (Carvalho et al. 2012; Utsunomia et al. 2014), *Ophisternon aenigmaticum* Rosen & Greenwood, 1976 (2n = 46, FN = 52) (Nirchio et al. 2011), and *O. bengalense* McClelland, 1844 (2n = 46, FN = 52) (Carvalho et al. 2012), as well as the species of family Mastacembelidae of the same order (2n = 48, FN = 58–88) (Khuda-Bukhsh and Barat 1987). The fundamental numbers of *M. albus* is reduced to 50% of norm in synbranchid fishes and teleosts, which suggests that the acrocentric chromosomes of *M. albus* may have been formed by repeated tandem fusion of the ancestral acrocentric chromosomes contained in the ancestral karyotype of Synbranchidae. However, the hybridization signal of (TTAGGG)n at interstitial telomeric sites (ITSs) that appears to be remnants of fusion or inversion (Srikulnath et al. 2009, 2011, 2015) was not found in any chromosomes of *M. albus* in this study (Fig. 2). Comparative chromosome mapping of Asian swamp eel with zebrafish (*Danio rerio* Hamilton, 1822) using human bacterial artificial chromosome (BAC) probes revealed the Asian swamp eel retains a number of gene copies found in tetrapods, while other teleosts underwent the third genome duplication (GD), leading to multiple copies of the genes (Yi et al. 2001, Zhou et al. 2002). This suggests that Asian swamp eel retained the genome composition before the event of the third GD that occurred in teleosts (Zhou et al. 2002). Molecular structure of the pericentromeric regions of chromosome 4 which were high GC-rich have evolved in a concerted manner with amplification of the 18S – 28S rRNA genes. However, the chromosomal locations of the 18S – 28S rRNA genes varied in *M. albus* individuals (Fig. 2d, e), a phenomanon also observed in Chinese population on pair of chromosome 3 and/or chromosome 7 (Ji et al. 2003). In other synbranchid fishes, the 18S – 28S rRNA genes are generally located on a pair of chromosome 1 and on a pair of medium-sized acrocentric chromosomes in *O. aenigmaticum* (Nirchio et al. 2011), as well as on several other chromosome pairs in various pattern of *S. marmoratus* (Utsunomia et al. 2014). These results suggest that chromosomal locations of the 18S – 28S rRNA genes considerably differ in Synbranchidae.

In this study, eight microsatellite repeat motifs [(CAG)_10_, (CAA)_10_, (CGG)_10_, (GAG)_10_, (AGAT)_8_, (ACGC)_8_, (AAAT)_8_, and (AAATC)_6_] were dispersedly mapped on different chromosomes (Fig. 3). This suggests that the amplification of several microsatellite repeat motifs has occurred independently in the genome of *M. albus*. Interestingly, the dispersion of the microsatellite repeat motifs signals was co-localized to *M. albus* chromosomes with *Rex* retroelements. A similar case was found in cichlid species *Cichla monoculus* Agassiz, 1831, *Pterophyllum scalare* Schultze, 1823, and *Symphysodon discus* Heckel, 1840 (Schneider et al. 2015). This suggests that both *Rex* retroelements and microsatellite repeat motifs have co-amplified in the evolutionary process of the genome of *M. albus*.

### Organization of *Rex* retroelements (*MALRex1*, *MALRex3*, and *MALRex6*) on *Monopterus albus* chromosomes

The diversity of chromosomal distribution for *Rex* retroelements (*Rex1*, *Rex3*, and *Rex6*) was found in teleosts (Table 4). Two major distinctive patterns were observed: (1) compartmentalization as found in pericentromeric, centromeric, or telomeric regions, and (2) uniform dispersion throughout the genome or along the chromosomes (Ozouf-Costaz et al. 2004). Chromosomal distribution of *Rex1*, *Rex3*, and *Rex6* were generally located in the specific region together as compartmentalization within each family/order (Table 4). In this study, although *MALRex1* was dispersed throughout the genome, this element was predominantly localized to pericentromeric regions of all chromosomes except for chromosomes 4 and 9. By contrast, strong hybridization signals of *MALRex3* were dispersed on five chromosome pairs, with weak signals on seven chromosome pairs, which implies that *MALRex3* were specifically amplified in chromosomal regions of *M. albus*.

**Table 4.** Chromosomal distribution of *Rex1*, *Rex3*, and *Rex6* in teleosts. “n.d.” means not described.

Order	Family	Species	Chrosmosomal distribution			Chromosome number	Reference
			*Rex1*	*Rex3*	*Rex6*		
**Characiformes**	Characidae	*Astyanax paranae*	dispersion	telomeric region	n.d.	2n = 50	Silva et al. 2014
		*Astyanax fasciatus*	n.d.	telomeric region	n.d.	2n = 46 – 48	Pansonato-Alves et al. 2013
**Siluriformes**	Loricariidae	*Hisonotus leucofrenatus*	dispersion	n.d.	n.d.	2n = 54	Ferreira et al. 2011
		*Hypostomus nigromaculatus*	dispersion	dispersion	dispersion	2n = 76	Pansonato-Alves et al. 2013
		*Pseudotocinclus tietensis*	dispersion	dispersion	n.d.	2n = 54	Ferreira et al. 2011
**Salmoniformes**	Salmonidae	*Coregonus albula*	pericentromeric region	n.d.	n.d.	2n = 80	Symonová et al. 2013
		*Coregonus fontanae*	pericentromeric region	n.d.	n.d.	2n = 80	Symonová et al. 2013
**Synbranchiformes**	Synbranchidae	*Monopterus albus*	pericentromeric region and insterstitial site	dispersion	dispersion	2n = 24	in this study
**Perciformes**	Latidae	*Lates calcarifer*	telomeric region	centromeric region	n.d.	2n = 48	Kuznetsova et al. 2014
	Cichlidae	*Astronotus ocellatus*	centromeric region	telomeric region	telomeric region	2n = 48	Schneider et al. 2013
		*Cichla kelberi*	centromeric region	centromeric region	dispersion	2n = 48	Teixeira et al. 2009
		*Cichla monoculus*	telomeric region	telomeric region	telomeric region	2n = 48	Schneider et al. 2013
		*Geophagus proximus*	telomeric region	telomeric region	telomeric region	2n = 48	Schneider et al. 2013
		*Hemichromis bimaculatus*	pericentromeric region	pericentromeric region	centromeric region	2n = 44	Valente et al. 2011
		*Melanochromis auratus*	pericentromeric region	pericentromeric region	pericentromeric region	2n = 44	Valente et al. 2011
		*Oreochromis niloticus*	pericentromeric region	pericentromeric region	pericentromeric region	2n = 44	Valente et al. 2011
		*Pterophyllum scalare*	centromeric region	telomeric region	telomeric region	2n = 48	Schneider et al. 2013
		*Oreochromis niloticus*	pericentromeric region	pericentromeric region	pericentromeric region	2n = 44	Valente et al. 2011
		*Symphysodon discus*	dispersion	telomeric region	telomeric region	2n = 60	Schneider et al. 2013
	Nototheniidae	*Dissostichus mawsoni*	dispersion	dispersion	n.d.	2n = 48	Ozouf-Costaz et al. 2004
		*Notothenia coriiceps*	dispersion	dispersion	n.d.	2n = 22	Ozouf-Costaz et al. 2004
		*Trematomus newnesi*	dispersion	dispersion	n.d.	2n = 46	Ozouf-Costaz et al. 2004
	Bathydraconidae	*Gymnodraco acuticeps*	dispersion	dispersion	n.d.	2n = 48	Ozouf-Costaz et al. 2004

The differences in the copy number and chromosomal distribution of *MALRex1*, *MALRex3*, and *MALRex6* suggest that these retroelements were independently amplified or lost in the lineage of *M. albus*, where *MALRex3* is prone to retain a copy number higher than *MALRex1* and *MALRex6*. A similar case of copy number variation in *Rex* retroelements was also found in several Antarctic nototheniid species (Ozouf-Costaz et al. 2004).

### Molecular diversity of *Rex* retroelements (*Rex1*, *Rex3*, and *Rex6*)

Three *Rex* retroelements were identified in the genome of *M. albus*, and the degree of sequence divergence for the three retroelements was high (14–67%) from other species in comparison. *MALRex1* and *MALRex3* showed high interspecific sequence divergences from Cyprinodontiformes and Characiformes, respectively, but low interspecific sequence divergences from Perciformes fishes for *Rex1* and Esociformes for *Rex3* (Suppl. materials 3 and 4). This suggests that *M. albus* and Perciformes or Esociformes shared relatively recent activity of *Rex1* or *Rex3*, respectively. The average K_s_/K_a_ value of *Rex1* was higher than 1 between all compared species and between *M. albus* and other species (Table 1). These results suggest that *Rex1* evolved under purifying selection and that retrotranspositions occurred during the evolution of teleosts. By contrast, the average K_s_/K_a_ value of *Rex3* was closer to 1, which suggests that after retrotransposition, *Rex3* was influenced by pseudogene-like evolution (Table 2) (McAllister et al. 1997).

Only few data of *Rex6* sequences were available because specific PCR primers were not feasibly effective to detect this element in the genome of teleosts (Volff et al. 2001, Ozouf-Costaz et al. 2004, Schneider et al. 2013). The absence of *Rex6* was observed in several Antarctic nototheniid species, but *Rex6* exists in some other species of the same order Perciformes (Volff et al. 2001, Ozouf-Costaz et al. 2004, Schneider et al. 2013). This suggests that *Rex6* might have rapidly diverged in teleosts. *MALRex6* showed high interspecific sequence divergences (approximately 60%) of *M. albus* from other teleosts (Suppl. material 5). This may indicate that the divergence of *Rex6* sequences of *M. albus* (or Synbranchidae in general) and other teleosts was rather ancestral. The average K_s_/K_a_ value of *Rex6* was less than 1 (Table 3). This suggests that *Rex6* has a more diverse function in teleosts.

The present results of chromosomal distribution and molecular diversity of four repetitive element groups (the 18S – 28S rRNA gene, telomeric sequences, microsatellite repeat motifs, and *Rex* retroelements) revealed the chromosome constitution and genome organization of Asian swamp eels. This enabled us to learn more about the chromosome constitution in synbranchid fishes and teleosts as a whole. Further work is required to investigate and compare synbranchid fishes, including *M. cuchia*, to better understand the process of karyotype and genome evolution in this lineage.

## Supplementary materials

Supplementary material 1 Supplementary Table 1 Link: https://doi.org/10.3897/CompCytogen.v11i3.11739.suppl1 Data type: Table Explanation note: Primers used molecular cloning in this study. This dataset is made available under the Open Database License (http://opendatacommons.org/licenses/odbl/1.0/). The Open Database License (ODbL) is a license agreement intended to allow users to freely share, modify, and use this Dataset while maintaining this same freedom for others, provided that the original source and author(s) are credited.Aorarat Suntronpong, Watcharaporn Thapana, Panupon Twilprawat, Ornjira Prakhongcheep, Suthasinee Somyong, Narongrit Muangmai, Surin Peyachoknagul, Kornsorn Srikulnath

Supplementary material 2 Supplementary Table 2 Link: https://doi.org/10.3897/CompCytogen.v11i3.11739.suppl2 Data type: Table Explanation note: Teleost species and nucleotide sequences of the *Rex1*, *Rex3*, and *Rex6* genes used in this study. “–” means no data. This dataset is made available under the Open Database License (http://opendatacommons.org/licenses/odbl/1.0/). The Open Database License (ODbL) is a license agreement intended to allow users to freely share, modify, and use this Dataset while maintaining this same freedom for others, provided that the original source and author(s) are credited.Aorarat Suntronpong, Watcharaporn Thapana, Panupon Twilprawat, Ornjira Prakhongcheep, Suthasinee Somyong, Narongrit Muangmai, Surin Peyachoknagul, Kornsorn Srikulnath

Supplementary material 3 Supplementary Table 3 Link: https://doi.org/10.3897/CompCytogen.v11i3.11739.suppl3 Data type: Table Explanation note: Pairwise comparison of nucleotide sequence divergences of *Rex1* among twenty eight teleosts. This dataset is made available under the Open Database License (http://opendatacommons.org/licenses/odbl/1.0/). The Open Database License (ODbL) is a license agreement intended to allow users to freely share, modify, and use this Dataset while maintaining this same freedom for others, provided that the original source and author(s) are credited.Aorarat Suntronpong, Watcharaporn Thapana, Panupon Twilprawat, Ornjira Prakhongcheep, Suthasinee Somyong, Narongrit Muangmai, Surin Peyachoknagul, Kornsorn Srikulnath

Supplementary material 4 Supplementary Table 4 Link: https://doi.org/10.3897/CompCytogen.v11i3.11739.suppl4 Data type: Table Explanation note: Pairwise comparison of nucleotide sequence divergences of *Rex3* among twenty eight teleosts. This dataset is made available under the Open Database License (http://opendatacommons.org/licenses/odbl/1.0/). The Open Database License (ODbL) is a license agreement intended to allow users to freely share, modify, and use this Dataset while maintaining this same freedom for others, provided that the original source and author(s) are credited.Aorarat Suntronpong, Watcharaporn Thapana, Panupon Twilprawat, Ornjira Prakhongcheep, Suthasinee Somyong, Narongrit Muangmai, Surin Peyachoknagul, Kornsorn Srikulnath

Supplementary material 5 Supplementary Table 5 Link: https://doi.org/10.3897/CompCytogen.v11i3.11739.suppl5 Data type: Table Explanation note: Pairwise comparison of nucleotide sequence divergences of *Rex6* among seventeen teleosts. This dataset is made available under the Open Database License (http://opendatacommons.org/licenses/odbl/1.0/). The Open Database License (ODbL) is a license agreement intended to allow users to freely share, modify, and use this Dataset while maintaining this same freedom for others, provided that the original source and author(s) are credited.Aorarat Suntronpong, Watcharaporn Thapana, Panupon Twilprawat, Ornjira Prakhongcheep, Suthasinee Somyong, Narongrit Muangmai, Surin Peyachoknagul, Kornsorn Srikulnath

Supplementary material 6 Supplementary Figure 1 Link: https://doi.org/10.3897/CompCytogen.v11i3.11739.suppl6 Data type: Image Explanation note: Phylogenetic placements of partial nucleotide sequences of *Rex1* from 28 teleosts and from *Physalaemus henselii*, Peters 1872 (KU842414) as the outgroup. Support values at each node are Bayesian posterior probability. This dataset is made available under the Open Database License (http://opendatacommons.org/licenses/odbl/1.0/). The Open Database License (ODbL) is a license agreement intended to allow users to freely share, modify, and use this Dataset while maintaining this same freedom for others, provided that the original source and author(s) are credited.Aorarat Suntronpong, Watcharaporn Thapana, Panupon Twilprawat, Ornjira Prakhongcheep, Suthasinee Somyong, Narongrit Muangmai, Surin Peyachoknagul, Kornsorn Srikulnath

Supplementary material 7 Supplementary Figure 2 Link: https://doi.org/10.3897/CompCytogen.v11i3.11739.suppl7 Data type: Image Explanation note: Phylogenetic placements of partial nucleotide sequences of *Rex6* from 17 teleosts and from *Podocnemis unifilis*, Troschel 1848 (KR336823) as the outgroup. Support values at each node are Bayesian posterior probability. This dataset is made available under the Open Database License (http://opendatacommons.org/licenses/odbl/1.0/). The Open Database License (ODbL) is a license agreement intended to allow users to freely share, modify, and use this Dataset while maintaining this same freedom for others, provided that the original source and author(s) are credited.Aorarat Suntronpong, Watcharaporn Thapana, Panupon Twilprawat, Ornjira Prakhongcheep, Suthasinee Somyong, Narongrit Muangmai, Surin Peyachoknagul, Kornsorn Srikulnath
