# Supplementary material for: Karyological characterization and identification of four repetitive element groups (the 18S – 28S rRNA gene, telomeric sequences, microsatellite repeat motifs, Rex retroelements) of the Asian swamp eel (Monopterus albus)
Source: Comp Cytogenet. 2017 Jun 22;11(3):435–62. doi: 10.3897/CompCytogen.v11i3.11739 (PMC5646660; doi:10.3897/CompCytogen.v11i3.11739)
Supplement: Supplementary material 1 — Supplementary Table 1 [file comparative_cytogenetics-11-435-s001.docx]

Supplementary Table 1. Primers used molecular cloning in this study

| Gene |  | Primer | Reference |
| --- | --- | --- | --- |
| 18S−28S rRNA | F | 5′-CCGCTTTGGTGACTCTTGAT-3′ | Teixeira et al. 2009 |
|  | R | 5′-CCGAGGACCTCACTAAACCA-3′ |  |
| *Rex1* | F | 5′-TTCTCCAGTGCCTTCAACACC-3′ | Volff et al. 2000. |
|  | R | 5′-TTCTCCAGTGCCTTCAACACC-3′ |  |
| *Rex3* | F | 5′-CGGTGAYAAAGGGCAGCCCTG-3′ | Volff et al. 1999. |
|  | R | 5′-TGGCAGACNGGGGTGGTGGT-3′ |  |
| *Rex6* | F | 5′-TAAAGCATACATGGAGCGCCAC-3′ | Volff et al. 2001. |
|  | R | 5′-GGTCCTCTACCAGAGGCCTGGG-3′ |  |
