# Supplementary material for: Karyological characterization and identification of four repetitive element groups (the 18S – 28S rRNA gene, telomeric sequences, microsatellite repeat motifs, Rex retroelements) of the Asian swamp eel (Monopterus albus)
Source: Comp Cytogenet. 2017 Jun 22;11(3):435–62. doi: 10.3897/CompCytogen.v11i3.11739 (PMC5646660; doi:10.3897/CompCytogen.v11i3.11739)
Supplement: Supplementary material 2 — Supplementary Table 2 [file comparative_cytogenetics-11-435-s002.doc]

Supplementary Table 2. Teleost species and nucleotide sequences of the *Rex1*, *Rex3*, and *Rex6* genes used in this study. “–” means no data.

| Order | Family | Species | Abbreviation | *Rex1* | *Rex3* | *Rex6* |
| --- | --- | --- | --- | --- | --- | --- |
| Anguilliformes | Anguillidae | *Anguilla anguilla* | AAN | − | AJ400457 | − |
|  |  | *Anguilla japonica* | AJA | AJ288465 | − | − |
| Cypriniformes | Cyprinidae | *Cyprinus carpio* | CCA | − | AJ400451 | − |
|  |  | *Danio rerio* | DRE | − | AJ400365 | − |
| Characiformes | Characidae | *Astyanax fasciatus* | AFA | − | KC832400 | − |
| Siluriformes | Loricariidae | *Corumbataia cuestae* | CCU | − | GQ505956 | − |
|  |  | *Hisonotus leucofrenatus* | HLE | GQ505952 | − | − |
|  |  | *Hypostomus nigromaculatus* | HNI | JX680315 | − | − |
|  |  | *Otocinclus flexilis* | OFL | GQ505951 | − | − |
|  |  | *Pseudotocinclus tietensis* | PTI | GQ505953 | GQ505954 | − |
| Salmoniformes | Salmonidae | *Coregonus albula* | CAL | JQ731754 | − | − |
| Escociformes | Esocidae | *Esox lucius* | ELU | − | AJ400447 | − |
| Beloniformes | Adrianichthyidae | *Oryzias javanicus* | OJA | − | − | − |
|  |  | *Oryzias latipes* | OLA | AJ288486 | AJ400435 | AJ293522 |
| Cyprinodontiformes | Fundulidae | *Fundulus* sp. | FUN | AJ288483 | AJ400377 | − |
|  | Poeciliidae | *Gambusia affinis* | GAF | AJ288434 | AJ400426 | AJ293532 |
|  |  | *Heterandria bimaculata* | HBI | − | AJ400424 | − |
|  |  | *Phallichthys amates* | PAM | AJ288440 | AJ400413 | − |
|  |  | *Poecilia formosa* | PFO | − | AJ400423 | AJ293536 |
|  |  | *Poecilia mexicana* | PME | AJ288454 | − | − |
|  |  | *Poeciliopsis gracilis* | PGR | AJ288431 | − | AJ293526 |
|  |  | *Xiphophorus maculatus* | XMA | AJ288451 | AJ400393 | AJ293517 |
| Synbranchiformes | Synbranchidae | *Monopterus albus* | MAL | LC110446 | LC110447 | LC110448 |
| Perciformes | Latidae | *Lates calcarifer* | LCA | KC842207 | − | − |
|  | Percichthyidae | *Siniperca chuatsi* | SCH | − | AJ400440 | − |
|  | Cichlidae | *Astronotus ocellatus* | AOC | JX576350 | JX576359 | JX576414 |
|  |  | *Cichla monoculus* | CMO | JX576350 | JX576355 | JX576404 |
|  |  | *Cichlasoma labridens* | CLA | AJ288470 | AJ400374 | AJ293549 |
|  |  | *Crenicichla* sp*.* | CSP | − | − | HM535301 |
|  |  | *Geophagus proximus* | GPR | JX576350 | − | JX576420 |
|  |  | *Geophagus surinamensis* | GSU | − | HM535302 | − |
|  |  | *Hemichromis bimaculatus* | HBI | AJ288481 | − | AJ293544 |
|  |  | *Melanochromis auratus* | MAU | − | − | HM535303 |
|  |  | *Oreochromis niloticus* | ONI | AJ288473 | AJ400371 | AJ293545 |
|  |  | *Pterophyllum scalare* | PSC | JX576342 | JX576373 | JX576427 |
|  |  | *Symphysodon discus* | SDI | JX576350 | JX576400 | JX576459 |
|  | Nototheniidae | *Dissostichus mawsoni* | DMA | AY331101 | − | − |
|  |  | *Notothenia coriiceps* | NCO | AY331095 | − | − |
|  |  | *Trematomus newnesi* | TNE | AY331098 | − | − |
|  | Bathydraconidae | *Gymnodraco acuticeps* | GAC | AY331099 | − | − |
|  | Gempylidae | *Rexea solandri* | RSO | − | − | EU263887 |
| Scorpaeniformes | Cottidae | *Battrachocottus baikalensis* | BBA | AJ288462 | AJ400361 | − |
