# Supplementary material for: Karyological characterization and identification of four repetitive element groups (the 18S – 28S rRNA gene, telomeric sequences, microsatellite repeat motifs, Rex retroelements) of the Asian swamp eel (Monopterus albus)
Source: Comp Cytogenet. 2017 Jun 22;11(3):435–62. doi: 10.3897/CompCytogen.v11i3.11739 (PMC5646660; doi:10.3897/CompCytogen.v11i3.11739)
Supplement: Supplementary material 3 — Supplementary Table 3 [file comparative_cytogenetics-11-435-s003.doc]

Supplementary Table 3. Pairwise comparison of nucleotide sequence divergences of *Rex1* among twenty eight teleosts.

|  | AJA | PTI | HLE | HNI | OFL | CAL | OLA | FUN | GAF | PME | PAM | PGR | XMA | MAL | LCA | AOC | CMO | CLA | GPR | HBI | ONI | PSC | SDI | DMA | NCO | TNE | GAC | BBA |
| --- | --- | --- | --- | --- | --- | --- | --- | --- | --- | --- | --- | --- | --- | --- | --- | --- | --- | --- | --- | --- | --- | --- | --- | --- | --- | --- | --- | --- |
| *Anguilla japonica*  (AJA) |  |  |  |  |  |  |  |  |  |  |  |  |  |  |  |  |  |  |  |  |  |  |  |  |  |  |  |  |
| *Pseudotocinclus tietensis* (PTI) | 0.2805 |  |  |  |  |  |  |  |  |  |  |  |  |  |  |  |  |  |  |  |  |  |  |  |  |  |  |  |
| *Hisonotus leucofrenatus* (HLE) | 0.2098 | 0.1561 |  |  |  |  |  |  |  |  |  |  |  |  |  |  |  |  |  |  |  |  |  |  |  |  |  |  |
| *Hypostomus nigromaculatus* (HNI) | 0.2390 | 0.2780 | 0.2268 |  |  |  |  |  |  |  |  |  |  |  |  |  |  |  |  |  |  |  |  |  |  |  |  |  |
| *Otocinclus flexilis* (OFL) | 0.2317 | 0.2561 | 0.2073 | 0.2341 |  |  |  |  |  |  |  |  |  |  |  |  |  |  |  |  |  |  |  |  |  |  |  |  |
| *Coregonus albula* (CAL) | 0.2488 | 0.2805 | 0.2366 | 0.2829 | 0.2634 |  |  |  |  |  |  |  |  |  |  |  |  |  |  |  |  |  |  |  |  |  |  |  |
| *Oryzias latipes (*OLA) | 0.2976 | 0.3585 | 0.3220 | 0.3000 | 0.322 | 0.2805 |  |  |  |  |  |  |  |  |  |  |  |  |  |  |  |  |  |  |  |  |  |  |
| *Fundulus* sp*.* (FUN) | 0.2683 | 0.3122 | 0.2780 | 0.2878 | 0.2780 | 0.3073 | 0.3439 |  |  |  |  |  |  |  |  |  |  |  |  |  |  |  |  |  |  |  |  |  |
| *Gambusia affinis* (GAF) | 0.3976 | 0.4244 | 0.3878 | 0.4000 | 0.3927 | 0.3902 | 0.4293 | 0.4073 |  |  |  |  |  |  |  |  |  |  |  |  |  |  |  |  |  |  |  |  |
| *Poecilia mexicana* (PME) | 0.4220 | 0.4610 | 0.4171 | 0.4317 | 0.4244 | 0.4146 | 0.4585 | 0.4537 | 0.1439 |  |  |  |  |  |  |  |  |  |  |  |  |  |  |  |  |  |  |  |
| *Phallichthys amates* (PAM) | 0.4195 | 0.4317 | 0.4073 | 0.4341 | 0.4098 | 0.4122 | 0.4537 | 0.4268 | 0.0854 | 0.1659 |  |  |  |  |  |  |  |  |  |  |  |  |  |  |  |  |  |  |
| *Poeciliopsis gracilis* (PGR) | 0.4171 | 0.4341 | 0.4244 | 0.4463 | 0.4220 | 0.4195 | 0.5000 | 0.4463 | 0.3976 | 0.4220 | 0.4098 |  |  |  |  |  |  |  |  |  |  |  |  |  |  |  |  |  |
| *Xiphophorus maculatus* (XMA) | 0.4049 | 0.4341 | 0.3927 | 0.4195 | 0.4073 | 0.3976 | 0.4463 | 0.4268 | 0.0659 | 0.1463 | 0.0927 | 0.4049 |  |  |  |  |  |  |  |  |  |  |  |  |  |  |  |  |
| *Monopterus albus* (MAL) | 0.1634 | 0.2707 | 0.2293 | 0.2537 | 0.2537 | 0.2780 | 0.3317 | 0.2927 | 0.3683 | 0.3951 | 0.3976 | 0.4195 | 0.3805 |  |  |  |  |  |  |  |  |  |  |  |  |  |  |  |
| *Lates calcarifer* (LCA) | 0.1707 | 0.2537 | 0.1732 | 0.2244 | 0.2268 | 0.2415 | 0.3146 | 0.2634 | 0.3976 | 0.4195 | 0.4293 | 0.4122 | 0.4024 | 0.1976 |  |  |  |  |  |  |  |  |  |  |  |  |  |  |
| *Astronotus ocellatus* (AOC) | 0.2415 | 0.3341 | 0.2878 | 0.2829 | 0.2805 | 0.3024 | 0.3341 | 0.3341 | 0.4024 | 0.4439 | 0.4439 | 0.4366 | 0.4171 | 0.1780 | 0.2829 |  |  |  |  |  |  |  |  |  |  |  |  |  |
| *Cichla monoculus* (CMO) | 0.2390 | 0.3317 | 0.2854 | 0.2805 | 0.2780 | 0.3000 | 0.3317 | 0.3366 | 0.4024 | 0.4439 | 0.4439 | 0.4341 | 0.4171 | 0.1756 | 0.2829 | 0.0024 |  |  |  |  |  |  |  |  |  |  |  |  |
| *Cichlasoma labridens* (CLA) | 0.2146 | 0.2780 | 0.2317 | 0.2463 | 0.2098 | 0.2683 | 0.3073 | 0.1902 | 0.3976 | 0.4220 | 0.4220 | 0.4195 | 0.4098 | 0.2122 | 0.2049 | 0.2659 | 0.2683 |  |  |  |  |  |  |  |  |  |  |  |
| *Geophagus proximus* (GPR) | 0.2415 | 0.3341 | 0.2878 | 0.2829 | 0.2805 | 0.3024 | 0.3341 | 0.3341 | 0.4024 | 0.4439 | 0.4439 | 0.4366 | 0.4171 | 0.1780 | 0.2829 | 0.0000 | 0.0024 | 0.2659 |  |  |  |  |  |  |  |  |  |  |
| *Heterandria bimaculata* (HBI) | 0.2244 | 0.2732 | 0.2341 | 0.2463 | 0.2293 | 0.2829 | 0.3122 | 0.1902 | 0.3902 | 0.4195 | 0.4073 | 0.4146 | 0.4000 | 0.2122 | 0.2317 | 0.2732 | 0.2756 | 0.1000 | 0.2732 |  |  |  |  |  |  |  |  |  |
| *Oreochromis niloticus* (ONI) | 0.2049 | 0.2659 | 0.2317 | 0.2415 | 0.2195 | 0.2683 | 0.3024 | 0.1756 | 0.3902 | 0.4220 | 0.4122 | 0.4366 | 0.4024 | 0.2049 | 0.2195 | 0.2512 | 0.2537 | 0.078 | 0.2512 | 0.0366 |  |  |  |  |  |  |  |  |
| *Pterophyllum scalare* (PSC) | 0.2390 | 0.3317 | 0.2854 | 0.2805 | 0.2780 | 0.3000 | 0.3317 | 0.3366 | 0.4024 | 0.4439 | 0.4439 | 0.4341 | 0.4171 | 0.1756 | 0.2829 | 0.0024 | 0.0000 | 0.2683 | 0.0024 | 0.2756 | 0.2537 |  |  |  |  |  |  |  |
| *Symphysodon discus* (SDI) | 0.2024 | 0.2610 | 0.2220 | 0.2317 | 0.2098 | 0.2634 | 0.3024 | 0.1854 | 0.3951 | 0.4146 | 0.4220 | 0.4268 | 0.4098 | 0.2073 | 0.2049 | 0.2537 | 0.2561 | 0.0341 | 0.2537 | 0.0829 | 0.0561 | 0.2561 |  |  |  |  |  |  |
| *Dissostichus mawsoni* (DMA) | 0.1829 | 0.2878 | 0.2415 | 0.2537 | 0.2610 | 0.2780 | 0.3439 | 0.2829 | 0.3927 | 0.4171 | 0.4317 | 0.4220 | 0.4024 | 0.1488 | 0.2098 | 0.2561 | 0.2537 | 0.2146 | 0.2561 | 0.2390 | 0.2268 | 0.2537 | 0.2049 |  |  |  |  |  |
| *Notothenia coriiceps* (NCO) | 0.1854 | 0.2951 | 0.2463 | 0.2537 | 0.2707 | 0.2854 | 0.3341 | 0.2854 | 0.3927 | 0.4220 | 0.4244 | 0.4195 | 0.4098 | 0.1488 | 0.2171 | 0.2634 | 0.2610 | 0.2122 | 0.2634 | 0.2366 | 0.2244 | 0.2610 | 0.2049 | 0.0366 |  |  |  |  |
| *Trematomus newnesi* (TNE) | 0.1902 | 0.2927 | 0.2463 | 0.2561 | 0.2610 | 0.2902 | 0.3415 | 0.2829 | 0.3902 | 0.4195 | 0.4244 | 0.4390 | 0.4049 | 0.1537 | 0.2317 | 0.2756 | 0.2732 | 0.2146 | 0.2756 | 0.2366 | 0.2244 | 0.2732 | 0.2049 | 0.0415 | 0.0488 |  |  |  |
| *Gymnodraco acuticeps* (GAC) | 0.1976 | 0.2951 | 0.2512 | 0.2707 | 0.2659 | 0.2976 | 0.3415 | 0.2927 | 0.3951 | 0.4220 | 0.4268 | 0.4341 | 0.4122 | 0.1610 | 0.2293 | 0.2732 | 0.2707 | 0.2244 | 0.2732 | 0.2512 | 0.2390 | 0.2707 | 0.2171 | 0.0463 | 0.0366 | 0.0488 |  |  |
| *Battrachocottus baikalensis* (BBA) | 0.1780 | 0.3098 | 0.2610 | 0.2829 | 0.2634 | 0.2756 | 0.3439 | 0.3171 | 0.4024 | 0.4317 | 0.4244 | 0.4293 | 0.4268 | 0.2293 | 0.2268 | 0.2902 | 0.2878 | 0.2512 | 0.2902 | 0.2732 | 0.2512 | 0.2878 | 0.2390 | 0.2268 | 0.2390 | 0.2341 | 0.2439 |  |
