# Supplementary material for: Karyological characterization and identification of four repetitive element groups (the 18S – 28S rRNA gene, telomeric sequences, microsatellite repeat motifs, Rex retroelements) of the Asian swamp eel (Monopterus albus)
Source: Comp Cytogenet. 2017 Jun 22;11(3):435–62. doi: 10.3897/CompCytogen.v11i3.11739 (PMC5646660; doi:10.3897/CompCytogen.v11i3.11739)
Supplement: Supplementary material 4 — Supplementary Table 4 [file comparative_cytogenetics-11-435-s004.doc]

Supplementary Table 4. Pairwise comparison of nucleotide sequence divergences of *Rex3* among twenty four teleosts.

|  | AAN | CCA | DRE | AFA | CCU | PTI | ELU | OLA | FUN | GAF | HBI | PFO | PAM | XHE | MAL | SCH | AOC | CMO | CLA | GSU | ONI | PSC | SDI | BBA |
| --- | --- | --- | --- | --- | --- | --- | --- | --- | --- | --- | --- | --- | --- | --- | --- | --- | --- | --- | --- | --- | --- | --- | --- | --- |
| *Anguilla anguilla* (AAN) |  |  |  |  |  |  |  |  |  |  |  |  |  |  |  |  |  |  |  |  |  |  |  |  |
| *Cyprinus carpio* (CCA) | 0.2682 |  |  |  |  |  |  |  |  |  |  |  |  |  |  |  |  |  |  |  |  |  |  |  |
| *Danio rerio* (DRE) | 0.3179 | 0.2119 |  |  |  |  |  |  |  |  |  |  |  |  |  |  |  |  |  |  |  |  |  |  |
| *Astyanax fasciatus* (AFA) | 0.6821 | 0.6656 | 0.6954 |  |  |  |  |  |  |  |  |  |  |  |  |  |  |  |  |  |  |  |  |  |
| Corumbataia cuestae (CCU) | 0.6523 | 0.6424 | 0.6689 | 0.3013 |  |  |  |  |  |  |  |  |  |  |  |  |  |  |  |  |  |  |  |  |
| *Pseudotocinclus tietensis* (PTI) | 0.6457 | 0.6291 | 0.6556 | 0.3344 | 0.1325 |  |  |  |  |  |  |  |  |  |  |  |  |  |  |  |  |  |  |  |
| *Esox lucius* (ELU) | 0.2781 | 0.1589 | 0.2119 | 0.6656 | 0.6391 | 0.6225 |  |  |  |  |  |  |  |  |  |  |  |  |  |  |  |  |  |  |
| *Oryzias latipes* (OLA) | 0.2715 | 0.1921 | 0.2086 | 0.6623 | 0.6391 | 0.6325 | 0.1854 |  |  |  |  |  |  |  |  |  |  |  |  |  |  |  |  |  |
| *Fundulus* sp*.*(FUN) | 0.3013 | 0.1987 | 0.2417 | 0.6457 | 0.6623 | 0.6457 | 0.1987 | 0.2086 |  |  |  |  |  |  |  |  |  |  |  |  |  |  |  |  |
| *Gambusia affinis* (GAF) | 0.3444 | 0.2285 | 0.2616 | 0.6358 | 0.6424 | 0.6192 | 0.2219 | 0.2219 | 0.1457 |  |  |  |  |  |  |  |  |  |  |  |  |  |  |  |
| *Heterandria bimaculata* (HBI) | 0.3113 | 0.1854 | 0.2483 | 0.6258 | 0.6291 | 0.6126 | 0.1987 | 0.2086 | 0.1258 | 0.1225 |  |  |  |  |  |  |  |  |  |  |  |  |  |  |
| *Poecilia Formosa* (PFO) | 0.3146 | 0.2252 | 0.2748 | 0.6424 | 0.6457 | 0.6291 | 0.2152 | 0.2252 | 0.1291 | 0.1192 | 0.1093 |  |  |  |  |  |  |  |  |  |  |  |  |  |
| *Phallichthys amates* (PAM) | 0.3013 | 0.1987 | 0.2417 | 0.6358 | 0.6358 | 0.6159 | 0.1821 | 0.1921 | 0.0993 | 0.0795 | 0.0828 | 0.0695 |  |  |  |  |  |  |  |  |  |  |  |  |
| *Xiphophorus hellerii* (XHE) | 0.2947 | 0.1921 | 0.2384 | 0.6291 | 0.6291 | 0.6093 | 0.1821 | 0.1854 | 0.0927 | 0.0762 | 0.0695 | 0.0629 | 0.0265 |  |  |  |  |  |  |  |  |  |  |  |
| *Monopterus albus* (MAL) | 0.3013 | 0.1887 | 0.2285 | 0.6656 | 0.6291 | 0.6291 | 0.1854 | 0.2252 | 0.2483 | 0.2748 | 0.2583 | 0.2748 | 0.2450 | 0.2318 |  |  |  |  |  |  |  |  |  |  |
| *Siniperca chuatsi* (SCH) | 0.2748 | 0.1689 | 0.2152 | 0.6689 | 0.6424 | 0.6358 | 0.1490 | 0.1722 | 0.1887 | 0.2152 | 0.1887 | 0.1921 | 0.1722 | 0.1623 | 0.1921 |  |  |  |  |  |  |  |  |  |
| *Astronotus ocellatus* (AOC) | 0.2616 | 0.2649 | 0.2881 | 0.6556 | 0.6556 | 0.6556 | 0.2682 | 0.2550 | 0.2550 | 0.2815 | 0.2583 | 0.2815 | 0.2450 | 0.2450 | 0.2881 | 0.2715 |  |  |  |  |  |  |  |  |
| *Cichla monoculus* (CMO) | 0.2351 | 0.2318 | 0.2682 | 0.6589 | 0.6556 | 0.6424 | 0.2152 | 0.2086 | 0.2152 | 0.2450 | 0.2351 | 0.2417 | 0.1987 | 0.1921 | 0.2616 | 0.2185 | 0.1093 |  |  |  |  |  |  |  |
| *Cichlasoma labridens* (CLA) | 0.2384 | 0.2185 | 0.2517 | 0.6457 | 0.6490 | 0.6358 | 0.2185 | 0.2219 | 0.1954 | 0.2152 | 0.2053 | 0.2152 | 0.1821 | 0.1722 | 0.2649 | 0.2086 | 0.1126 | 0.0795 |  |  |  |  |  |  |
| *Geophagus surinamensis* (GSU) | 0.6523 | 0.6126 | 0.6623 | 0.2980 | 0.1722 | 0.2252 | 0.6358 | 0.6258 | 0.6358 | 0.6225 | 0.6291 | 0.6291 | 0.6159 | 0.6026 | 0.6126 | 0.6291 | 0.6325 | 0.6258 | 0.6225 |  |  |  |  |  |
| *Oreochromis niloticus* (ONI) | 0.2185 | 0.1788 | 0.2483 | 0.6556 | 0.6325 | 0.6325 | 0.1921 | 0.2086 | 0.1854 | 0.2053 | 0.1887 | 0.2086 | 0.1722 | 0.1589 | 0.2318 | 0.1887 | 0.1556 | 0.1192 | 0.1026 | 0.6159 |  |  |  |  |
| *Pterophyllum scalare* (PSC) | 0.3278 | 0.3179 | 0.3311 | 0.6623 | 0.6523 | 0.6490 | 0.3079 | 0.3179 | 0.3245 | 0.3311 | 0.3046 | 0.3344 | 0.3013 | 0.2980 | 0.3344 | 0.3245 | 0.1954 | 0.1755 | 0.1755 | 0.6325 | 0.2318 |  |  |  |
| *Symphysodon discus* (SDI) | 0.2417 | 0.2285 | 0.2583 | 0.6589 | 0.6656 | 0.6457 | 0.2252 | 0.2285 | 0.1954 | 0.2384 | 0.2185 | 0.2252 | 0.1954 | 0.1821 | 0.2781 | 0.2252 | 0.1258 | 0.0795 | 0.0430 | 0.6391 | 0.1093 | 0.1921 |  |  |
| *Battrachocottus baikalensis* (BBA) | 0.2616 | 0.2252 | 0.2815 | 0.6523 | 0.6325 | 0.6258 | 0.2318 | 0.2086 | 0.2285 | 0.2450 | 0.2384 | 0.2583 | 0.2185 | 0.2152 | 0.2748 | 0.2020 | 0.2318 | 0.1854 | 0.1755 | 0.6192 | 0.1788 | 0.2914 | 0.1921 |  |
