# Supplementary material for: Karyological characterization and identification of four repetitive element groups (the 18S – 28S rRNA gene, telomeric sequences, microsatellite repeat motifs, Rex retroelements) of the Asian swamp eel (Monopterus albus)
Source: Comp Cytogenet. 2017 Jun 22;11(3):435–62. doi: 10.3897/CompCytogen.v11i3.11739 (PMC5646660; doi:10.3897/CompCytogen.v11i3.11739)
Supplement: Supplementary material 5 — Supplementary Table 5 [file comparative_cytogenetics-11-435-s005.doc]

Supplementary Table 5. Pairwise comparison of nucleotide sequence divergences of *Rex6* among seventeen teleosts.

|  | OLA | GAF | PFO | PGR | XMA | MAL | AOC | CLA | CMO | CRE | GPR | HBI | MAU | ONI | PSC | SDI | RSO |
| --- | --- | --- | --- | --- | --- | --- | --- | --- | --- | --- | --- | --- | --- | --- | --- | --- | --- |
| *Oryzias latipes* (OLA) |  |  |  |  |  |  |  |  |  |  |  |  |  |  |  |  |  |
| *Gambusia affinis* (GAF) | 0.1645 |  |  |  |  |  |  |  |  |  |  |  |  |  |  |  |  |
| *Poecilia Formosa* (PFO) | 0.1436 | 0.0809 |  |  |  |  |  |  |  |  |  |  |  |  |  |  |  |
| *Poeciliopsis gracilis* (PGR) | 0.1436 | 0.0601 | 0.0836 |  |  |  |  |  |  |  |  |  |  |  |  |  |  |
| *Xiphophorus maculatus* (XMA) | 0.1567 | 0.0522 | 0.0809 | 0.0339 |  |  |  |  |  |  |  |  |  |  |  |  |  |
| *Monopterus albus* (MAL) | 0.6423 | 0.6292 | 0.6188 | 0.6214 | 0.6240 |  |  |  |  |  |  |  |  |  |  |  |  |
| *Astronotus ocellatus* (AOC) | 0.2219 | 0.2376 | 0.2219 | 0.2219 | 0.2324 | 0.6266 |  |  |  |  |  |  |  |  |  |  |  |
| *Cichlasoma labridens* (CLA) | 0.2219 | 0.2376 | 0.2324 | 0.2272 | 0.2298 | 0.6162 | 0.1488 |  |  |  |  |  |  |  |  |  |  |
| *Cichla monoculus* (CMO) | 0.2037 | 0.2115 | 0.2063 | 0.2037 | 0.1984 | 0.6214 | 0.1462 | 0.1514 |  |  |  |  |  |  |  |  |  |
| *Crenicichla* sp*.* (CRE) | 0.1775 | 0.1906 | 0.1906 | 0.1802 | 0.1828 | 0.6162 | 0.1279 | 0.1175 | 0.0627 |  |  |  |  |  |  |  |  |
| *Geophagus proximus* (GPR) | 0.2742 | 0.2689 | 0.2742 | 0.2637 | 0.2637 | 0.6031 | 0.1958 | 0.2037 | 0.1410 | 0.1305 |  |  |  |  |  |  |  |
| *Hemichromis bimaculatus* (HBI) | 0.1514 | 0.0705 | 0.0313 | 0.0731 | 0.0705 | 0.6240 | 0.2193 | 0.2298 | 0.2010 | 0.1802 | 0.2689 |  |  |  |  |  |  |
| *Melanochromis auratus* (MAU) | 0.2037 | 0.2219 | 0.2010 | 0.2037 | 0.2037 | 0.6319 | 0.1567 | 0.1645 | 0.1227 | 0.1149 | 0.1906 | 0.2037 |  |  |  |  |  |
| *Oreochromis niloticus* (ONI) | 0.2219 | 0.2298 | 0.2063 | 0.2141 | 0.2141 | 0.6266 | 0.1619 | 0.1828 | 0.1410 | 0.1384 | 0.2063 | 0.2115 | 0.0444 |  |  |  |  |
| *Pterophyllum scalare* (PSC) | 0.2115 | 0.2272 | 0.2167 | 0.2141 | 0.2167 | 0.6240 | 0.1462 | 0.1619 | 0.0836 | 0.0809 | 0.1540 | 0.2115 | 0.1436 | 0.1593 |  |  |  |
| *Symphysodon discus* (SDI) | 0.2454 | 0.2428 | 0.2376 | 0.2350 | 0.2350 | 0.6345 | 0.1828 | 0.1671 | 0.1097 | 0.1149 | 0.1802 | 0.2376 | 0.1697 | 0.1802 | 0.1253 |  |  |
| *Rexea solandri* (RSO) | 0.6345 | 0.6345 | 0.6266 | 0.6188 | 0.6136 | 0.6554 | 0.6345 | 0.6319 | 0.6371 | 0.6084 | 0.6449 | 0.6188 | 0.6214 | 0.6292 | 0.6345 | 0.6475 |  |
