# Supplementary figures and images for: Karyological characterization and identification of four repetitive element groups (the 18S – 28S rRNA gene, telomeric sequences, microsatellite repeat motifs, Rex retroelements) of the Asian swamp eel (Monopterus albus)
Source: Comp Cytogenet. 2017 Jun 22;11(3):435–62. doi: 10.3897/CompCytogen.v11i3.11739 (PMC5646660; doi:10.3897/CompCytogen.v11i3.11739)

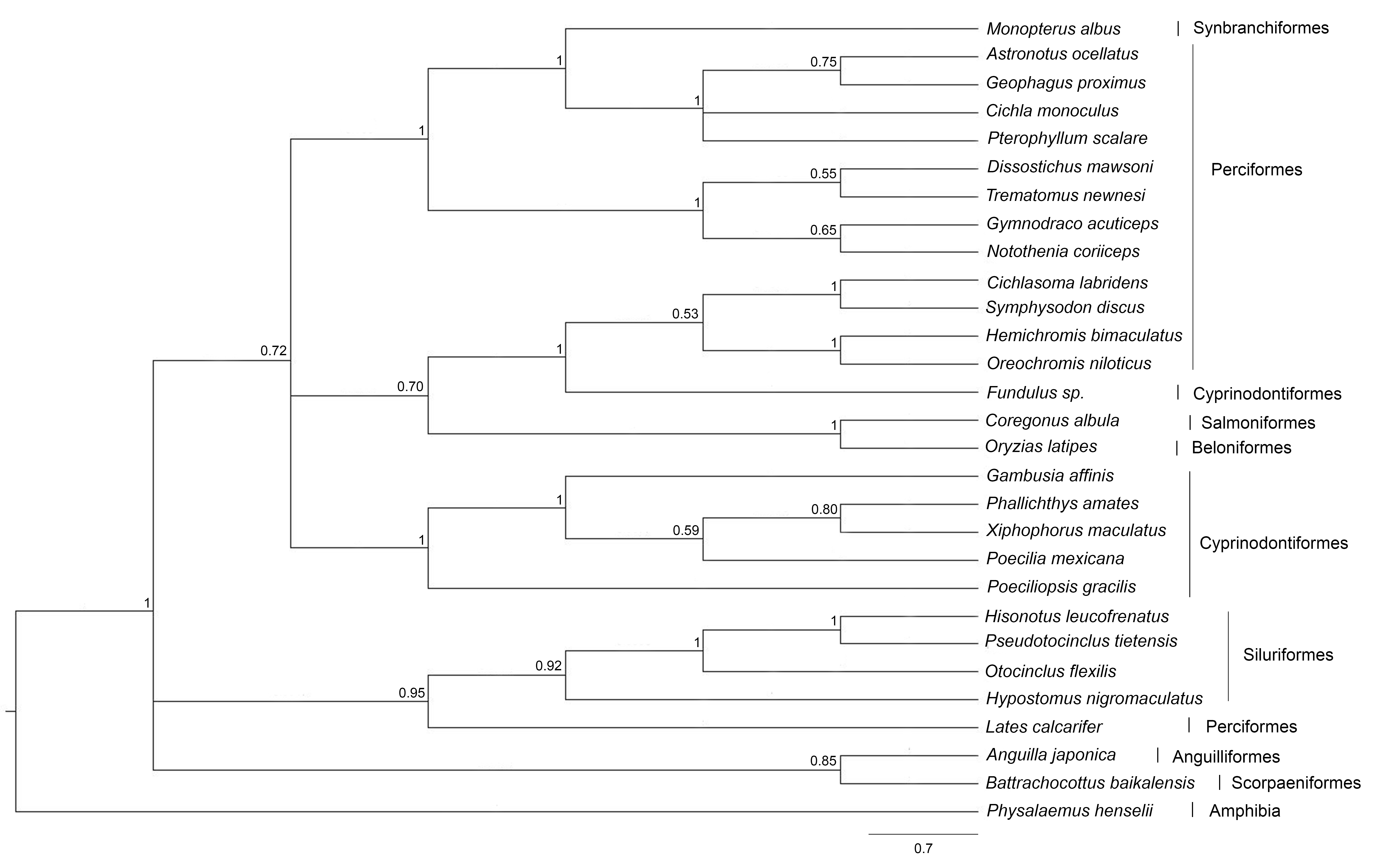

Supplement: Supplementary material 6 — Supplementary Figure 1 [file comparative_cytogenetics-11-435-s006.jpg]

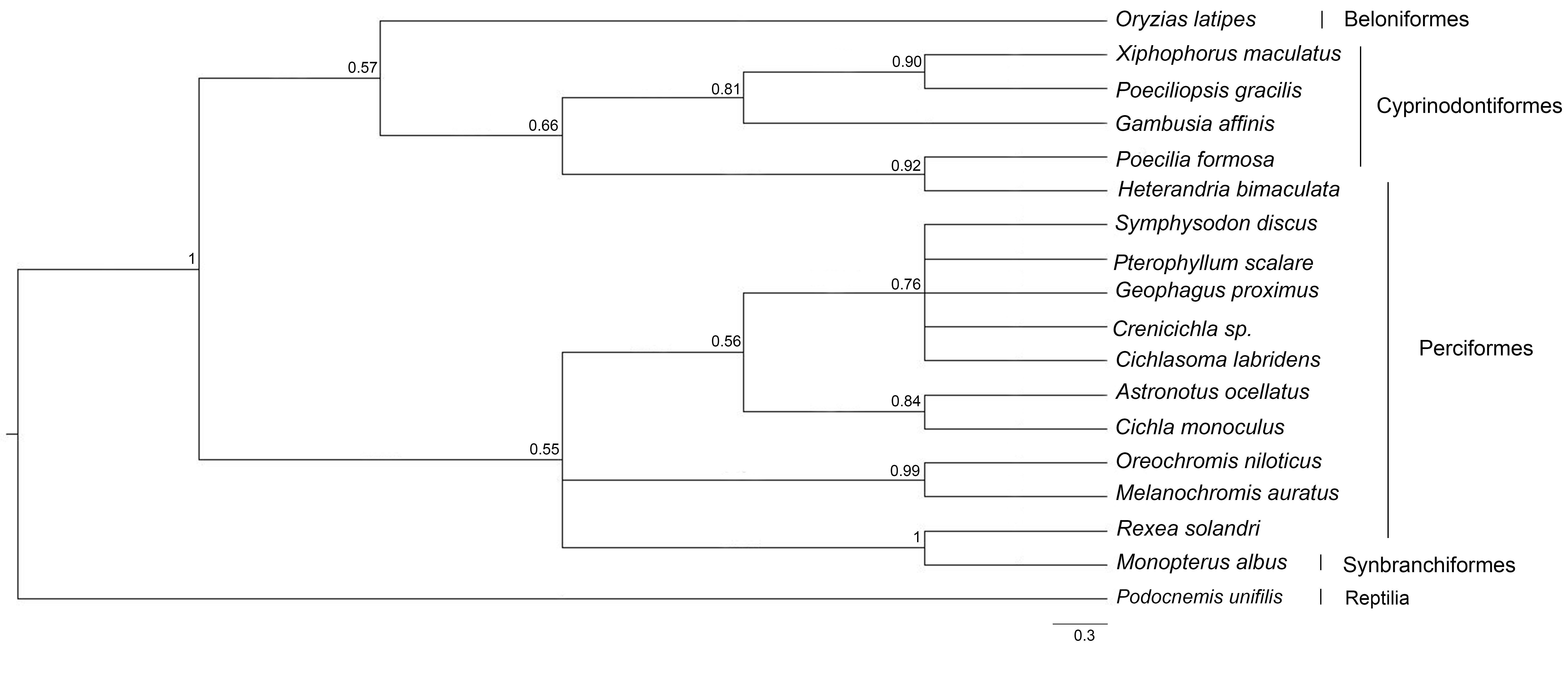

Supplement: Supplementary material 7 — Supplementary Figure 2 [file comparative_cytogenetics-11-435-s007.jpg]
